# Identification of Effective Subdominant Anti-HIV-1 CD8+ T Cells Within Entire Post-infection and Post-vaccination Immune Responses

**DOI:** 10.1371/journal.ppat.1004658

**Published:** 2015-02-27

**Authors:** Gemma Hancock, Hongbing Yang, Elisabeth Yorke, Emma Wainwright, Victoria Bourne, Alyse Frisbee, Tamika L. Payne, Mark Berrong, Guido Ferrari, Denis Chopera, Tomas Hanke, Beatriz Mothe, Christian Brander, M. Juliana McElrath, Andrew McMichael, Nilu Goonetilleke, Georgia D. Tomaras, Nicole Frahm, Lucy Dorrell

**Affiliations:** 1 Nuffield Department of Medicine, University of Oxford, Oxford, United Kingdom; 2 Oxford NIHR Biomedical Research Centre, University of Oxford, Oxford, United Kingdom; 3 Faculty of Medicine, University of Toronto, Toronto, Canada; 4 Department of Sexual Health, Royal Berkshire NHS Foundation Trust, Reading, United Kingdom; 5 Departments of Molecular Genetics and Microbiology, Surgery, Immunology, and Duke Human Vaccine Institute, Duke University, Durham, North Carolina, United States of America; 6 Institute of Infectious Diseases and Molecular Medicine & Division of Medical Virology, University of Cape Town, Cape Town, South Africa; 7 Irsicaixa AIDS Research Institute—HIVACAT, Hospital Germans Trias i Pujol, Badalona, Spain; 8 Institució Catalana de Recerca i Estudis Avançats (ICREA), Barcelona, Spain; 9 Vaccine and Infectious Diseases Division, Fred Hutchinson Cancer Research Center, Seattle, Washington, United States of America; 10 Department of Microbiology and Immunology, University of North Carolina School of Medicine, Chapel Hill, North Carolina, United States of America; University of Wisconsin, UNITED STATES

## Abstract

Defining the components of an HIV immunogen that could induce effective CD8+ T cell responses is critical to vaccine development. We addressed this question by investigating the viral targets of CD8+ T cells that potently inhibit HIV replication in vitro, as this is highly predictive of virus control in vivo. We observed broad and potent ex vivo CD8+ T cell-mediated viral inhibitory activity against a panel of HIV isolates among viremic controllers (VC, viral loads <5000 copies/ml), in contrast to unselected HIV-infected HIV Vaccine trials Network (HVTN) participants. Viral inhibition of clade-matched HIV isolates was strongly correlated with the frequency of CD8+ T cells targeting vulnerable regions within Gag, Pol, Nef and Vif that had been identified in an independent study of nearly 1000 chronically infected individuals. These vulnerable and so-called “beneficial” regions were of low entropy overall, yet several were not predicted by stringent conservation algorithms. Consistent with this, stronger inhibition of clade-matched than mismatched viruses was observed in the majority of subjects, indicating better targeting of clade-specific than conserved epitopes. The magnitude of CD8+ T cell responses to beneficial regions, together with viral entropy and HLA class I genotype, explained up to 59% of the variation in viral inhibitory activity, with magnitude of the T cell response making the strongest unique contribution. However, beneficial regions were infrequently targeted by CD8+ T cells elicited by vaccines encoding full-length HIV proteins, when the latter were administered to healthy volunteers and HIV-positive ART-treated subjects, suggesting that immunodominance hierarchies undermine effective anti-HIV CD8+ T cell responses. Taken together, our data support HIV immunogen design that is based on systematic selection of empirically defined vulnerable regions within the viral proteome, with exclusion of immunodominant decoy epitopes that are irrelevant for HIV control.

## Author Summary

Attempts to develop an HIV vaccine that elicits potent cell-mediated immunity have so far been unsuccessful. This is due in part to the use of immunogens that appear to recapitulate responses induced naturally by HIV that are, at best, partially effective. We previously showed that the capacity of CD8+ T cells from patients to block HIV replication in culture is strongly correlated with HIV control in vivo, therefore, we investigated the virological determinants of potent CD8+ T cell inhibitory activity. We observed that CD8+ T cells from patients with naturally low plasma viral loads (viremic controllers) were better able to inhibit the replication of diverse HIV strains in vitro than CD8+ T cells from HIV-noncontroller patients. Importantly, we also found that the potency of the antiviral activity in the latter group was strongly correlated with recognition of selected regions across the viral proteome that are critical to viral fitness. Vaccines that encode full-length viral proteins rarely elicited responses to these vulnerable regions. Taken together, our results provide insight into the characteristics of effective cell-mediated immune responses against HIV and how these may inform the design of better immunogens.

## Introduction

Only two HIV vaccines designed to elicit protective T cell responses have reached clinical efficacy testing, both with disappointing results [1][2][3]. The reasons for this are not completely understood, despite much accumulated knowledge regarding the characteristics of cell-mediated immune responses associated with HIV and SIV control. The limited magnitude and breadth of vaccine-induced T cell responses, particularly when compared with responses to similar vaccines in non-human primate models, the modest cytotoxic capacity of CD8+ T cells, waning of responses over time, bias towards targeting of more variable regions of the viral proteome and the modest immunogenicity of the vaccine vector regimens are all likely contributing factors [2][4][5][6][7][8]. A critical first step towards addressing this is to determine whether the antiviral efficacy of CD8+ T cells is a function of their specificity.

The HVTN 502 (Step) and 503 (Phambili) trials were a test-of-concept for induction of protective T cell responses that collectively evaluated Merck’s trivalent adenovirus type 5 HIV-1 Gag/Pol/Nef vaccine in ∼3800 subjects at high risk of HIV acquisition [1][9]. Post-hoc analyses of HVTN 502 have shown that individuals in whom vaccine-induced responses targeted ≥3 epitopes in Gag achieved a lower viral load after HIV infection than subjects without Gag responses; it is striking, however, that these subjects were a small minority among the vaccinees (<7%) [6]. While this confirms several observational studies that showed an association between HIV control and preferential recognition of Gag epitopes [10][11], the question remains as to why vaccines that express full-length Gag proteins have so far failed to induce responses that can impact on HIV replication after infection. The answer may be two-fold: first, immunodominance hierarchies of the T cell responses elicited by these vaccines often mimic those of natural infection, with ‘hotspots’ in variable and least vulnerable regions of the viral proteome [12]; second, even within Gag and other conserved proteins, not all epitopes are equal in terms of vulnerability to immune pressure, or ‘fragility’, which is defined by the capacity to maintain function in the face of genetic mutations [13]. Thus, the efficacy of cell-mediated immune responses may depend on the specific epitopes targeted, both within and outside Gag. This was demonstrated in an observational study of 950 clade B- and C-infected individuals, in whom responses to overlapping peptides (OLP) spanning the entire viral proteome were systematically analysed [14]. A ‘protective ratio’ (PR) was calculated for each OLP from the ratio of the median viral load in subjects who failed to respond to the OLP to responders. OLP with a protective ratio >1 were defined as ‘beneficial’. Of note, Gag proteins contained the majority of the beneficial regions, though not all of them, and also contained regions that were not targeted by protective responses. Together, these data support the ‘decoy’ hypothesis, which proposes that certain epitopes within the viral proteome elicit dominant yet irrelevant responses that serve to undermine effective targeting of regions of vulnerability [15]. This question will only be adequately addressed by clinical testing of rationally designed immunogens based on ‘beneficial’ regions, as proposed by Rolland et al. and Mothe et al. [15][14].

Aside from identifying specific beneficial targets, the precise mechanisms and effector functions of antiviral T cell responses that underlie heterogeneity in HIV control among infected individuals need to be defined. We showed in a prospective study that CD8+ T cell viral inhibitory activity in vitro strongly correlated with HIV control in vivo, reflected in both viral load set-point and CD4+ cell decline over time [16]. This indicates that CD8+ T cell viral inhibitory activity is expressed on a continuum and is not a discrete function that is unique to HIV controllers with protective HLA alleles, providing scope for induction of effective CD8+ T cell responses by vaccination of subjects who do not have a favourable genotype. Viral inhibition assays that use polyclonal T cell populations provide a composite measure of lytic and non-lytic activity of all circulating HIV-specific CD8+ T cells, which may be heterogeneous in their functional capacity [17][18][19][20][21][22]. This activity is detectable in acute infection in a minority but rapidly wanes, likely as a result of viral escape and / or functional impairment [23][24][21][25]. Low level activity has also been detected in HIV-naïve recipients of DNA and adenovirus type 5-vectored vaccines encoding full-length HIV proteins even though such vaccines are capable of eliciting substantial numbers of Gag- and Pol-specific cytokine-secreting T cells [23][26][3]. These observations underscore the need for better understanding of the factors that determine the potency of CD8+ T cell viral inhibitory activity.

We also showed previously that CD8+ T cell viral inhibition in chronically infected individuals did not correlate with the total magnitude of IFN-γ-positive T cell response to any single HIV protein, including Gag [16]. This was surprising, given the known associations between Gag responses and HIV control, and led us to propose the hypothesis that potent viral inhibition depends on preferential targeting of selected regions that are not limited to Gag nor predicted by conservation score alone. We hypothesised that responses to such critical regions are generally subdominant and that this may explain the lack of efficacy of T cell-inducing vaccines. To this end, we investigated CD8+ T cell-mediated inhibitory activity in a subset of HIV-positive HVTN 502 and 503 vaccine trial participants. This comprised recipients of both the vaccine and placebo who were sampled at the same time during early HIV infection (1 year). They were naïve to antiretroviral therapy (ART), with CD4 cell counts >350 cells/μl, and were not selected for low virus loads or protective HLA class I alleles. In parallel, we studied ART-naïve subjects who showed spontaneous long-term control of HIV, with plasma viral loads consistently <5000 copies/ml (viremic controllers, VC). They were sampled later in infection (median 4.5 years) and were included as a reference cohort, as potent CD8+ T cell antiviral activity has been reported in such individuals [23][26][16].

## Results

### Limited potency and breadth of CD8+ T cell antiviral inhibitory activity in the majority of HIV-positive vaccine and placebo recipients

**Fig 1:**
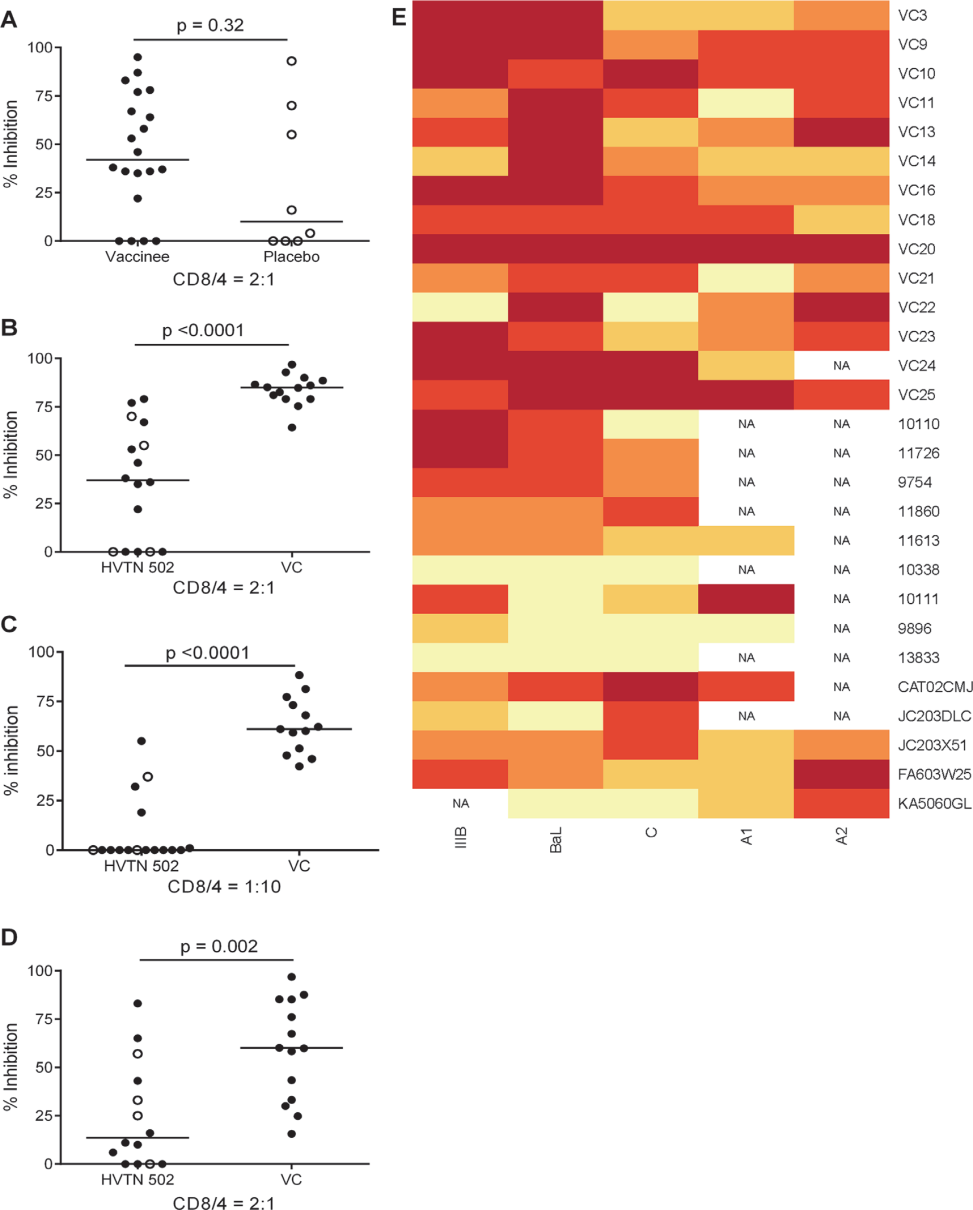
CD8+ T cell antiviral inhibitory activity in HIV-positive HVTN subjects and HIV viremic controllers. **A.** CD8+ T cell mediated-inhibition of a clade-matched virus was measured in 28 HIV-positive HVTN 502 & 503 subjects at a CD8+/CD4+ cell ratio of 2:1. Eight subjects are not shown as cell recoveries allowed testing at a CD8+/CD4+ cell ratio of 1:1 only (n = 6, see S1 Table) or CD4+ cells did not survive HIV superinfection (n = 2). Data are stratified by vaccine or placebo allocation. **B-D.** CD8+ T cells from 16 HVTN 502 participants and 14 VC (all infected with clade B viruses) were tested for inhibition of a clade B HIV isolate at CD8+/CD4+ cell ratios of 2:1 (**B**) and 1:10 (**C**) or inhibition of a clade C isolate, ES X-1936 (CD8+/CD4+ = 2:1) (**D**). Placebos are indicated by open circles. In all graphs, horizontal lines indicate medians. **E.** Heatmap showing potency and breadth of CD8+ T cell-mediated inhibition (CD8+/CD4+ cells = 2:1) among 14 viremic controllers and 14 HIV-positive HVTN 502 and 503 participants for whom at least 3 virus isolates were tested. Viral inhibition was measured on day 6 for all viruses except A2 (RW93024), for which it was measured on day 3 due its different replication kinetics (see Methods). Darker colour indicates higher inhibition; scale 0–100%, grading—20%.

CD8+ T cell antiviral activity was measured in 34 HIV-positive HVTN 502 & 503 trial participants, who were infected with clade B and C viruses respectively. They were aligned for duration of infection, early post-infection viral load and CD4+ cell counts. Only a minority had either a protective HLA class I allele (n = 7, 20%) or evidence of spontaneous viremia control, indicated by plasma viral loads consistently below 5000 copies/ml (n = 5, 15%) (Table 1). We included both vaccinees and placebos in order to maximise the number of subjects with samples available for analysis. Fourteen VC with viral loads <5000 copies/ml were studied in parallel as a reference cohort. The estimated duration of HIV infection in latter ranged from 1–11 years. Six (43%) had a protective HLA class I allele and all were presumed clade B-infected (Table 2). The inclusion of clade B and C cohorts enabled us to ascertain whether the association between CD8+ T cell inhibitory activity and HIV control was clade-independent, as suggested by our previous results [16]. However, a major goal of this study was to explore the extent of cross-clade inhibition (breadth) using a panel of laboratory-adapted and primary HIV isolates representing clades A, B and C strains, as this had not been systematically examined in HIV-positive individuals before. CD8+ T cells from HIV-positive HVTN 502 & 503 participants were tested according to PBMC availability, using at least one clade B and one clade C virus, while all VC were tested against five viral isolates (S1 Table). Among the HIV-positive trial participants in whom viral inhibitory activity against a clade-matched virus was analysed at a CD8+/CD4+ cell ratio of 2:1, it was not significantly different between vaccinees (n = 20) and placebo (n = 8) recipients (ranges 0–87% vs. 0–93%, p = 0.32; Fig. 1A). Because no difference was observed, analyses presented in the main were performed by combining data from both the vaccinee and placebo groups. However, the vaccinees were also analysed independently as they accounted for two-thirds of the HVTN cohorts. The data are shown in Supplementary Results, S1 Text. Whether data were combined or independent, the results were similar. Inhibition of a clade-matched virus was significantly higher among VC at CD8+/CD4+ T cell ratios of both 2:1 (medians 85% and 37% respectively, p <0.0001) (Fig. 1B) and 1:10 (medians 61% and 0% respectively, p <0.0001 (Fig. 1C). VC also showed more potent cross-clade inhibition than HVTN 502 participants when tested using a clade C virus (CD8+/CD4+ T cell ratio of 2:1—medians 60% vs. 14%, p = 0.002) (Fig. 1D). These differences remained significant when the placebos were excluded from the analyses (Supplementary Results, S1 Text). Cross-clade activity was analysed further using at least 3 viruses in 14 HVTN 502 & 503 participants and 14 VC. Differences in the potency and breadth of CD8+ T cell-mediated inhibitory responses in these groups are highlighted in the heatmap (Fig. 1E).

**Table 1:** Characteristics of HVTN 502 and 503 trial participants.

		502	503	p value
N		20	16	
Sex, male (%)		100	31	-
Age, years		30 (23–35)	23 (22–28)	0.07
Known duration of infection (days)		364 (363–365)	370 (364–375)	0.10
CD4+ T cell count, cells/μl*		633 (487–671)	559 (463–651)	0.46
Plasma viral load at 3 months, log_10_ copies/ml		4.2 (3.3–4.7)	4.4 (3.6–5.2)	0.31
Known protective alleles (n) ^§^		4	3	-
Clade				
	B	19/20	0/16	
	C	0/20	13/16	
	Other	1/20	2/16 (CA, CB)	
	Not defined	0/20	1/16	

Data shown are median values (interquartile range) unless otherwise indicated.

* At time of sampling

^§^ HLA-B*27, B51, B*5701/03, B*5801 and B*8101

**Table 2:** Characteristics of Viremic Controller subjects.

N	14
Sex, male (%)	40
Age, years—median (IQR)	45 (37–49)
Known duration of infection, years* - median (IQR)	4.5 (2.25–6.25)
CD4+ T cell count, cells/μl* - median (IQR)	721 (672–885)
Plasma viral load, log_10_ copies/ml* - median (IQR)	3.1 (2.9–3.3)
Known protective alleles, n ^§^	6
Clade—B^#^	14/14

* At time of sampling

^§^ HLA-B*27, B51, B*5701/03, B*5801 and B*8101

^#^ Presumed clade due to geographical location

We have previously reported a significant inverse relationship between CD8+ T cell antiviral activity measured 6 months post-infection in a primary HIV infection cohort and viral load set-point, a known predictor of the rate of progression to AIDS [16]. In the present study, CD8+ T cell inhibitory activity was measured later. Nevertheless, there was still a significant inverse correlation between CD8+ T cell inhibition of a clade-matched virus and viral load set-point (which was attained within 100 days of infection in the HVTN trial participants) or current viral load in the VC (r = -0.49, p = 0.0009, S1 Fig.).

### CD8+ T cell antiviral potency is strongly associated with targeting of known ‘beneficial’ regions

**Fig 2:**
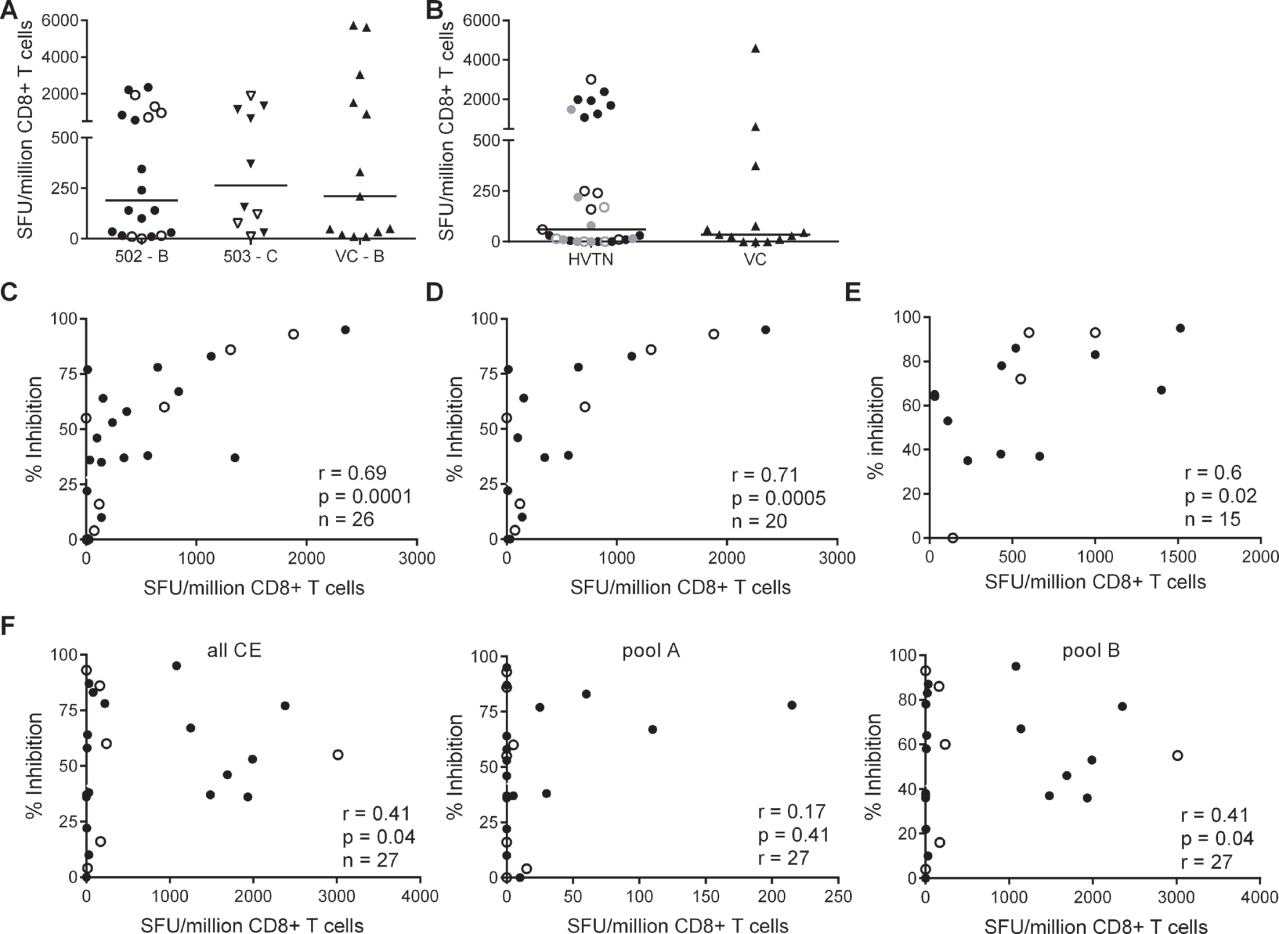
CD8+ T cell inhibitory activity and targeting of beneficial regions and conserved elements within the HIV proteome. CD8+ T cell responses to peptides based on (**A**) clade-specific ‘beneficial’ regions and (**B**) Gag ‘conserved elements’ were measured by IFN-γ Elispot assays. Net responses (background subtracted) are shown; values for negative controls were median (IQR)– 10 (0–15) SFU/million CD8+ T cells. Horizontal lines indicate median values. HVTN vaccinees and placebos are shown as closed and open symbols respectively in **A**. In **B**, HVTN subjects are grouped together and represented as follows: HVTN 502—vaccinees, black closed circles, placebos, black open circles; HVTN 503—vaccinees, grey closed circles, placebos, grey open circles. VC are shown as triangles in **A & B**. Six HVTN 503 subjects were excluded as viral subtype data were not confirmed at the time of the analysis. One VC subject was excluded as no sample was available for Elispot assay. **C.** Correlation between CD8+ T cell inhibition of a clade-matched virus (CD8+/CD4+ cell ratio = 2:1) and magnitude of CD8+ T cell responses to beneficial peptides (summed) in 26 HVTN subjects. **D.** The analysis was repeated after removal of subjects with protective HLA class I alleles and (**E**) with short-term cell lines expanded from CD8+ T cells recovered from Elispot assays in 15 subjects that were then tested with individual peptides from the pools which elicited a response in the ex vivo Elispot assay. **For C-F**: closed circles—502 and 503 vaccinees; open circles—502 and 503 placebos. **F.** Correlation between CD8+ T cell inhibition (2:1 ratio) of a clade-matched virus and magnitude of CD8+ T cell responses to conserved elements peptides in 27 HVTN subjects (left panel—sum of all CE peptides, middle—CE pool A, right—CE pool B).

The finding that HIV-positive trial participants showed less potent inhibition of a clade-matched virus isolate than VC was consistent with results from previous studies of early infected individuals [16][24]. Here, we extended these observations to clade-mismatched viruses. The broader CD8+ T cell inhibitory responses in VC suggested that they preferentially recognised conserved viral epitopes. However, when examining responses within the groups, we observed more potent inhibition of clade-matched than mismatched viruses in VC and HIV-positive trial participants alike. This indicated that CD8+ T cells targeting clade-specific viral epitopes must contribute to the overall potency of the response. To investigate this further, we used ex vivo IFN-γ Elispot assays to measure the magnitude of responses to two sets of overlapping 15-mer peptides. The first corresponded to the beneficial regions that were defined by Mothe et al. in clade B and clade C-infected populations (S2 and S3 Tables) and the second to a set of ‘conserved elements’ (CE) peptides that were originally defined by Rolland et al. and consisted of 7 regions in Gag p24 (S4 Table) [14][27][28]. The peptides representing beneficial regions were constituted in pools according to their previously defined protective ratio, with the first pool of each protein containing the peptides with the highest protective ratio (higher number indicating lower viral load in responders compared with non-responders) [14]. CE peptides were divided into pools A & B, also in accordance with previously observed associations with low virus loads [29] (S4 Table). To match their infecting clade, VC and HVTN 502 participants were tested with a peptide set representing beneficial regions in Clade B and HVTN 503 subjects were tested with the Clade C beneficial peptide set. All three groups were tested with the same CE peptide set. For all Elispot assays, CD8+ T cells were obtained from the same sample as that used in the viral inhibition assay (except for 2 VC in whom it was necessary to use an additional sample obtained within 1 year of the original bleed). Summed frequencies of IFN-γ-producing CD8+ T cells targeting the beneficial and CE peptides are shown in Fig. 2. The median response to beneficial peptides was 190 and 262 SFU/million CD8+ T cells for HVTN 502 and 503 groups respectively and 210 SFU/million CD8+ T cells for the VC (Fig. 2A). The median response to the CE peptides was 60 SFU/million CD8+ T cells for the combined HVTN groups and 35 SFU/million CD8+ T cells for the VC (Fig. 2B). These differences were not statistically significant, nor were there significant differences between vaccinees and placebos in terms of the magnitude of response to either beneficial (medians 198 vs. 415 SFU/million CD8+ T cells, p = 0.99) or CE peptides (medians 55 vs 60 SFU/million CD8+ T cells, p = 0.6).

This group of VC did not show significantly higher responses to beneficial or CE peptides than the HVTN subjects. This was unexpected in the light of previous reports but likely reflected the longer duration of infection (median 4.5 years vs. 1 year), which may be associated with loss of responses to epitopes within the regions studied, due to mutational escape [14][29][30][31][32]. For example, the two VC who were HLA-B*5701-positive did not make detectable responses to the beneficial or CE peptide pools that contained immunodominant Gag epitopes restricted by this allele (TW10 and KF11).

We next explored the relationship between virus inhibition and the magnitude of CD8+ T cell responses to the beneficial and CE regions in the HVTN subjects. We observed a strong correlation between the magnitude of T cell responses to beneficial regions and CD8+ T cell-mediated inhibition of a clade-matched virus (r = 0.69, p = 0.0001 for a CD8+/CD4+ cell ratio of 2:1) (Fig. 2C). This relationship was also confirmed using a lower CD8+/CD4+ cell ratio of 1:1 (r = 0.5, p = 0.01) and importantly, was maintained after removal of subjects with protective HLA class I alleles (HLA-B*27, B51, B*5701/03, B*5801, B*81) (r = 0.71, p = 0.0005) (Fig. 2D). Furthermore, these correlations remained statistically significant after exclusion of placebos (Supplementary Results, S1 Text). Taken together, these analyses suggested that CD8+ T cell viral inhibition of >85% (i.e. the median response in VC) was associated with a beneficial peptide response threshold of ∼1300 SFU/million CD8+ T cells. Additional support for the relationship between CD8+ T cell viral inhibition and magnitude of T cell responses to beneficial regions was obtained in a subset of subjects (n = 15) in which individual peptides were tested in cultured Elispot assays. The highest viral inhibition also correlated with the higher magnitude T cell responses to individual beneficial peptides (r = 0.61, p = 0.02, Fig. 2E). Unexpectedly, there was a weaker association between the magnitude of T cell responses to the conserved elements pools and CD8+ T cell viral inhibition (r = 0.41, p = 0.04) (Fig. 2F). This positive relationship was also maintained after exclusion of placebos (Supplementary Results, S1 Text) and was largely driven by responses to the conserved elements pool B, containing peptides spanning CE 4, 5 and 6.

**Fig 3:**
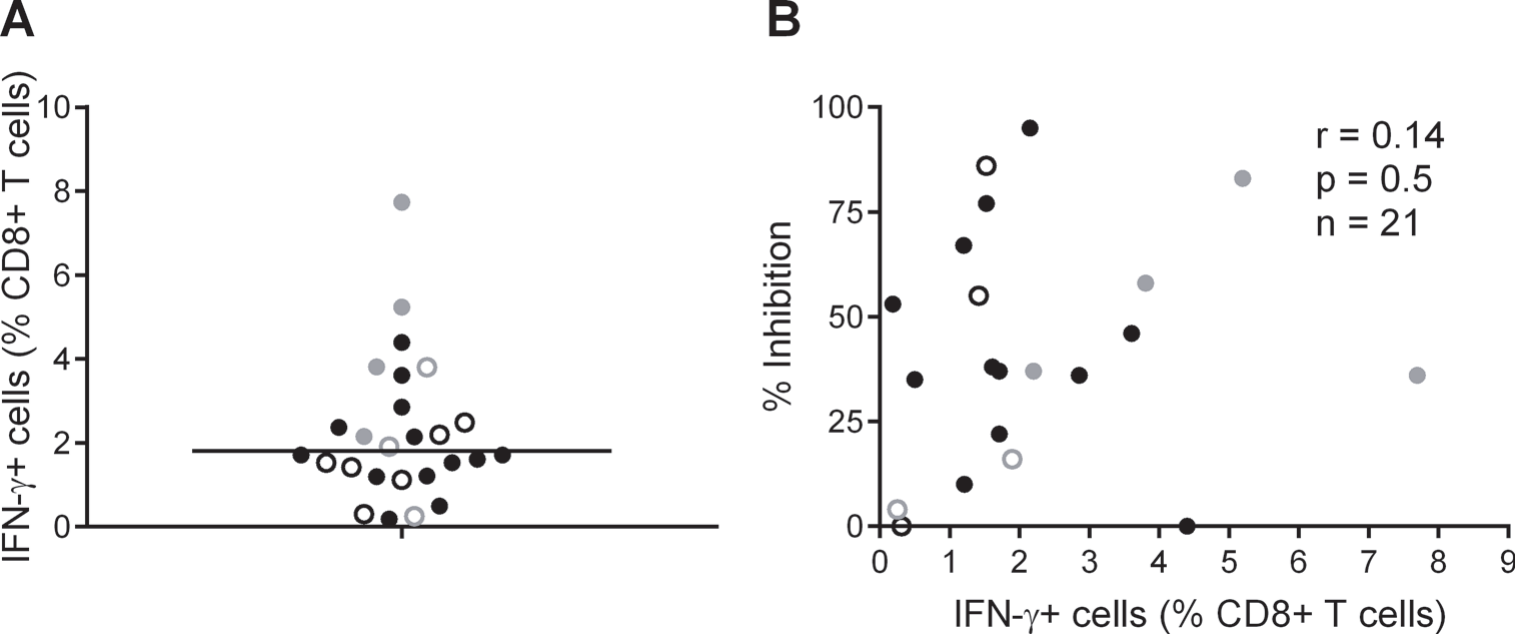
CD8+ T cell responses to PTE peptides based on to the entire HIV proteome were measured in HIV-positive HVTN participants (502—vaccinees, black closed circles; placebos, black open circles) and (503—vaccinees, grey closed circles; placebos, grey open circles) at week 5 post-infection by intracellular staining for IFN-γ. **A.** **B.** Correlation between CD8+ T cell inhibition (CD8+/CD4+ cell ratio = 2:1) of a clade-matched virus and magnitude of CD8+ T cell response to the entire HIV proteome, colour-coded as indicated in A. Five subjects are not shown in B, due to lack of viral inhibition data for the 2:1 ratio.

We also analysed the frequency of T cell responses to the total HIV proteome as these had been measured previously by intracellular staining for IFN-γ (at a median of 5 weeks after HIV infection) after stimulation of PBMC with clade B consensus potential T cell epitope (PTE) peptide sets [2][30]. These were selected to optimise the detection of CD8+ T cell responses to circulating viruses and thus ensure accurate measurement of the maximum response [33]. The total proteome response (median) was 1.81% CD8+ T cells (Fig. 3A), with no significant difference between HVTN 502/503 vaccinees and placebos (median 2.1% and 1.5% of CD8+ T cells respectively, p = 0.23), which is similar to data obtained from chronic infection cohorts [31]. There was no correlation between CD8+ T cell antiviral activity and responses to the whole proteome, either for HVTN subjects as a whole (r = 0.14, p = 0.5) (Fig. 3B) or for the vaccinees only (Supplementary Results, S1 Text).

### Variation in CD8+ T cell antiviral activity is best explained by the magnitude of responses to beneficial peptides

In view of the strong correlation between CD8+ T cell antiviral activity and recognition of beneficial peptides, we explored this relationship further using a series of univariate and multivariable regression models, with CD8+ T cell antiviral activity as the dependent variable. We investigated associations with the following independent variables: 1) the Shannon entropy score for each beneficial region as a measure of its variability at the population level; 2) the magnitude of responses to beneficial regions (‘total beneficial’ response); 3) the magnitude of the Gag component of the beneficial regions (‘beneficial Gag’ response), in order to ascertain how much this contributed to the total beneficial response; 4) the magnitude of responses to CE peptides; 5) the ratio of magnitude of responses to beneficial regions to the total proteome response (relative magnitude or immunodominance) and 6) the presence of protective (‘good’) or non-protective (‘bad’) HLA class I alleles.

For our first set of models, entropy was used as the primary independent (or predictor) variable of interest since both the beneficial regions and CE regions were largely derived from conserved, i.e. low entropy regions in the viral proteome [14][28]. Thus, our first regression model included entropy as the only independent variable. Total beneficial responses, beneficial Gag responses or CE responses were then each added separately to this baseline model to ascertain whether they improved the fit of the model (as captured by a change in the model r^2^) and, thus, whether they were independently associated with CD8+ T cell activity. Entropy alone explained 13.5% of the variance in inhibition. Addition of the total beneficial response or the beneficial Gag response each improved the fit of the model (by 46% and 24% respectively) and the contribution of each of these was statistically significant (Table 3). By contrast, addition of the CE response had no effect (increase in model r^2^ of 0.1%).

**Table 3:** Univariate linear regression models to investigate associations between entropy, beneficial and conserved elements responses and % inhibition (dependent variable).

	Model 1	Model 2	Model 3	Model 4
Entropy	152.8	144.2	126.8	142.8
	[-146.7–452.3]	[31.1–257.3]	[15.1–268.7]	[-99.1–384.7]
	p = 0.08	p = 0.02	p = 0.09	p = 0.08
Total beneficial response	-	0.03	-	-
		[0.02–0.04]		
		**p <0.001**		
Beneficial Gag response	-	-	0.03	-
			[0.01–0.05]	
			**p = 0.01**	
Conserved elements response	-	-	-	0.001
				[-0.02–0.02]
				p = 0.91
Model r^2^	0.135	0.594	0.372	0.136

Values shown are parameter estimates [95% confidence intervals] for each model.

All models: entropy refers to Shannon entropy score for beneficial peptides

Model 2: total beneficial response = sum of responses to beneficial peptide pools

Model 3: beneficial Gag response = sum of responses to beneficial Gag pools only

Model 4: conserved elements (CE) response = sum of responses to CE pools

We also constructed three multivariable regression models that included various combinations of the following factors: magnitude of total beneficial responses, relative magnitude or entropy of beneficial regions and good or bad HLA class I alleles. The combinations of covariates for these models were chosen to allow us to investigate several potential pathways for any associations, based on hypothesised interactions between absolute and relative magnitude of responses and between certain HLA alleles and entropy of epitopes restricted by these alleles. These models explained 39–49% of the variance in CD8+ T cell inhibition and all were significant as a whole. However, in each case the magnitude of the response to beneficial regions made the strongest unique contribution whereas the contribution of the other variables was not statistically significant (Table 4).

**Table 4:** Multivariate linear regression models to investigate associations between beneficial responses, entropy, HLA alleles and % inhibition (dependent variable).

	Parameter estimate	95% CI	P
**Model 1**			
Constant	4.97	-33.1–43	0.78
Magnitude	0.03	-0.004–0.06	0.08
Entropy	125.42	-47.7–299	0.14
Ratio of protective / total response	92.55	-359.9–545	0.67
Model r^2^	0.39		
**Model 2**			
Constant	5.87	-26–37.7	0.70
Magnitude	0.03	0.01–0.05	**0.003**
Entropy	130.89	-22.8–284.6	0.09
Good allele*	-2.72	-29.5–24	0.83
Model r^2^	0.47		
**Model 3**			
Constant	4.44	-27.1–35.9	0.77
Magnitude	0.03	0.01–0.05	**0.002**
Entropy	121.9	-21.9–265.7	0.09
Bad Allele^§^	9.25	-14.4–32.9	0.42
Model r^2^	0.49		

In each model, magnitude = total beneficial response (sum of responses to beneficial peptide pools)

Ratio of protective / total response = total beneficial response / total proteome response (both defined as IFN-γ+ cells/million CD8+ T cells)

* HLA-B*27, B*51, B*5701, B*5801, B*81

^§^ HLA-B*35 (Py), HLA-B*53

### Responses to vaccines encoding full-length HIV proteins are skewed towards non-beneficial regions of the viral proteome

**Fig 4:**
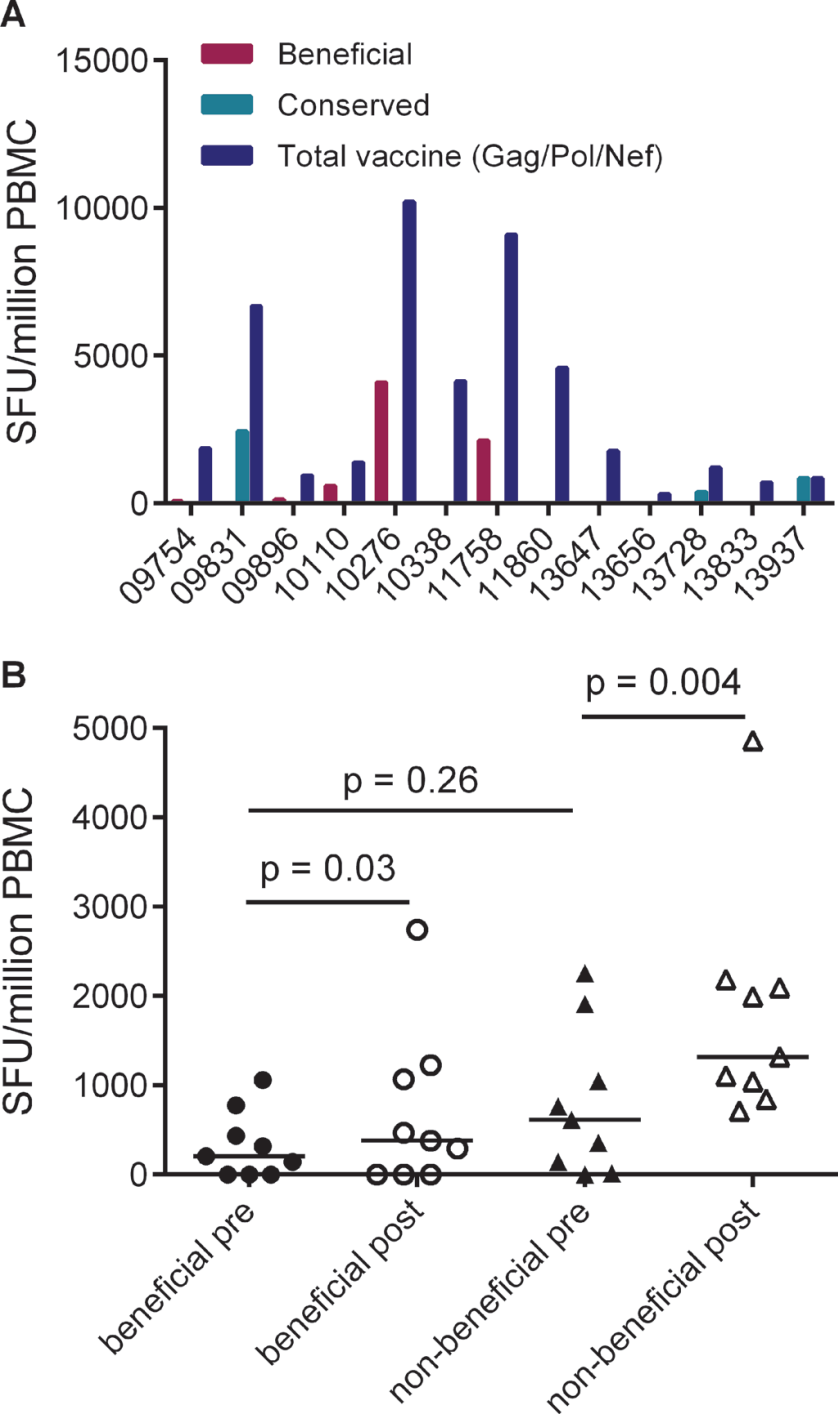
Vaccines based on full-length protein immunogens elicit subdominant responses to beneficial regions and / or conserved elements within the HIV proteome. **A.** Post-vaccination, pre-infection CD8+ T cell responses to overlapping peptides spanning the MRK Ad5 Gag/Pol/Nef immunogen determined by IFN-γ Elispot assay in 13 HVTN 502 participants (dark blue bars) (4 weeks post-second vaccination). Responses to beneficial regions and Gag conserved elements within the immunogen are indicated by magenta and turquoise bars respectively. **B.** Pre- and post-vaccination responses to beneficial and non-beneficial regions within Gag determined by IFN-γ Elispot assay in 9 HIV-positive subjects after therapeutic vaccination with MVA.HIVA.

Given that responses to beneficial regions were subdominant in HIV-infected individuals, we next investigated whether this was also the case for responses that are primed in HIV-naïve individuals by vaccines encoding full-length HIV proteins. Data on responses that developed post-vaccination and prior to HIV acquisition were available for 13/20 of the HVTN 502 trial participants in this analysis (sampled 4 weeks after the second vaccination) [6]. We compared the magnitudes of vaccine-induced responses to peptides spanning the entire Gag/Pol/Nef immunogen with beneficial and CE regions. Vaccination induced responses to beneficial regions in 5/13 patients and to CE regions 3/13 patients, while no response to any of these regions was detected in 5 subjects. Overall, vaccine-induced responses to beneficial regions accounted for a median (range) of 0% (0–43%) of the response to the entire immunogen in these subjects, despite representing 36% of the immunogen sequence (Fig. 4A).

Finally, we investigated whether natural immunodominance hierarchies were maintained or altered following the administration of a Gag immunogen as a therapeutic vaccine in chronic HIV infection. We mapped T cell responses to beneficial and non-beneficial regions before and after vaccination with an immunogen, ‘HIVA’ comprising full-length Gag p24/p17 sequences fused to a multiepitope string, delivered as a modified vaccinia virus Ankara-vectored vaccine to chronic ART-treated HIV-positive subjects with suppressed viremia [34][35]. Epitope mapping was performed in 9 subjects using overlapping 15-mers spanning p24 and p17, together with optimal 8–10-mer peptides for epitopes that had been defined previously (Table 5) [36]. We confined our analysis to responses to the Gag component of the immunogen, since the epitope string was, by definition, designed to focus responses on selected regions of the proteome. Prior to vaccination, the magnitude of summed responses to beneficial regions was lower than for non-beneficial Gag regions, although the difference was not statistically significant (median 205 and 615 SFU/million PBMC respectively, p = 0.27). MVA.HIVA vaccination significantly boosted T cell responses to the beneficial Gag regions (median change +150 SFU/million PBMC, p = 0.03). However, responses to non-beneficial Gag regions were preferentially expanded (median change +845 SFU/million PBMC, p = 0.004) (Fig. 4B, Table 5). Taken together, these data suggest that vaccines encoding full- or near full-length HIV proteins mimic natural HIV infection by eliciting responses that are biased towards non-beneficial targets, regardless of whether they are administered to HIV-naïve or primed individuals.

**Table 5:** Distribution of responses to Gag peptides in HIV-positive MVA.HIVA vaccinees.

	*Peptide in beneficial region in Gag*			*Peptide in non-beneficial region in Gag*		
Subject	Response(SFU/10^6^ PBMC)	Pre	Post	Response(SFU/10^6^ PBMC)	Pre	Post
001	SLFNTVATL	775	1070	AEQATQEVKNW	460	1090
				KDTKEALDKIEEIQN	115	715
004	WASRELERF	10	90			
006	SLFNTVATL	205	295	LNKIVRMYSPV	995	1010
				SVLSGGKLDAWEKIR	915	1085
009	KIRLRPGGK	520	1690	SPRTLNAW	115	360
	ACQGVGGPGHK	540	1055			
010				LVQNANPDCKSILRA	15	710
012	KIRLRPGGKKKYRLK	145	380	LVQNANPDCKSILRA	105	320
				GDIYKRWII	1210	2445
018				TQEVKNWMTETTLVQ	70	20
021	SLFNTVATL	120	180			
	GGKKKYKL	200	290			
022	VRMYSPVSILDIRQG	185	595	ISPRTLNAW	985	1750
				FRDYVDRFFK	250	630

## Discussion

The lack of a reliable correlate of protective immunity against HIV is a significant obstacle to systematic evaluation of vaccine candidates. Consequently, efforts to develop a T cell-based vaccine have focused broadly on recapitulating the immunological phenotype of HIV controllers, using immunogens incorporating near-complete gene sequences for many proteins. Recently, there has been greater emphasis on rationally designed immunogens, in particular, those that aim to maximise coverage of variable viral epitopes (mosaics) or avoid them altogether (conserved regions) [15][37][38][39][8]. CD8+ T cell-mediated viral inhibition was found to correlate with the frequency of T cells targeting conserved epitopes in HIV-uninfected vaccinees [8][40]. However, no vaccine candidate has yet been shown to elicit viral inhibitory activity of similar potency to that observed in HIV controllers. Here, we report that the total viral inhibitory capacity of anti-HIV CD8+ T cells is highly dependent on their specificity and we provide a mechanism to explain why conventional HIV immunogens elicit largely ineffective CD8+ T cell responses.

We reported previously that ex vivo CD8+ T cell-mediated viral inhibitory activity is inversely correlated with viral load set-point; we confirmed this finding here in genetically unrelated cohorts infected with different viruses [16]. While this is consistent with well-established associations between primary CD8+ T cell responses to HIV-1 and control of acute viraemia [41][42][32][43], the time interval between attainment of viral load set-point and sampling for the viral inhibition assay was longer in the present study, thus we cannot rule out the possibility that early control of viraemia was the cause rather than the consequence of the level of antiviral activity. It is also conceivable that a viral inhibition ‘set-point’ is attained soon after infection; this could explain the findings of Lecuroux et al., who reported that most HIV-infected individuals showed modest CD8+ T cell inhibitory activity throughout acute and early infection [24]. Nevertheless, our data give insight into the level of inhibitory activity that might be used as a benchmark to assess vaccine candidates: for example, inhibition of a clade-matched virus by ≥ 85% (observed in 50% of VC subjects but only 7% of HVTN trial participants) was associated with a median viral load of ∼ 2000 copies/ml. This suggests that the bar must be set very high if such assays are to be used to identify vaccine strategies that could clear HIV infection or reduce viral loads to undetectable levels [44].

We report for the first time, to our knowledge, that the breadth of inhibitory activity, indicated by inhibition of clade-mismatched viruses, was significantly greater in VC than subjects with uncontrolled viraemia. This suggested two non-mutually exclusive explanations: enrichment of the HIV-specific repertoire in VC for T cells recognising conserved epitopes and / or high frequencies of circulating cross-reactive CD8+ T cells that can tolerate epitope variation. However, potent clade-specific viral inhibitory activity, together with differential inhibition of diverse viruses was evident in both study groups. This led us to hypothesise that factors other than epitope conservation must play a role in the control of viral replication. We found that CD8+ T cell antiviral activity in HVTN subjects was highly correlated with the frequency of CD8+ T cells targeting selected peptides that had been shown in an independent study of two large cohorts to associate with control of viraemia [14]. This correlation was independent of protective HLA class I alleles, which suggests that effective CD8+ T cell responses may be restricted by a broader range of HLA class I alleles than previously suspected, as was also proposed by Mothe et al [14]. While the viral regions that were defined as beneficial were predominantly of low entropy, our regression analysis indicated that the magnitude of these responses accounted for a significantly greater proportion of the variation in viral inhibition than entropy alone. The Gag component of these regions explained nearly two-thirds of the effect. Interestingly, T cell responses to conserved elements peptides were weakly correlated with viral inhibition and this effect was driven by only three of the seven conserved regions tested. This is consistent with other studies showing that high population-level conservation per se does not necessarily predict viral fitness and may reflect the presence of invariant regions that are immunologically inert [27][45]. Collectively, these observations are not only reconcilable with previously described associations between broad Gag-specific T cell responses and reduced viral loads at the population level but also point to a mechanism that could explain them with greater precision [10][14][6]. The greater the breadth of responses to Gag, the higher the probability of targeting the most vulnerable epitopes, even though there is also the possibility of targeting the non-beneficial regions. The lack of responses to beneficial regions in some of the VC studied is quite likely explained by the small sample size studied and / or the extended time of untreated HIV infection which may have led to elimination of some of these T cell responses, or possibly that these VC made responses to other critical epitopes that were not represented in our peptide sets [32][46][47]. However, this does raise questions as to how long the effect of responses to beneficial regions lasts, in the face of ongoing viral escape. The rate of escape from CD8+ T cell responses is determined by the net effect on viral fitness of all escape mutations and is significantly slower in chronic than acute infection [48]. The association between the prevalence of T cell responses to beneficial regions and population-level viral load was made in chronically infected cohorts and suggests, therefore, that even though these beneficial responses may drive viral escape, the net effect is an overall impairment of viral fitness. This is consistent with observations made by Boutwell et al. who showed that CD8+ T cell escape mutations in HIV-1 Gag frequently impair viral fitness; many of the susceptible epitopes in their study were located in the beneficial regions [49].

It is possible that we have overlooked functional characteristics of Gag-specific CD8+ T cells such as the capacity to produce multiple cytokines simultaneously, as these have also been associated with control of viraemia [50][51]. However, viral inhibition assays arguably provide the most direct and complete measure of antiviral function, whereas the cytokines that are typically detected in assays of T cell polyfunctionality provide an indirect assessment. Our analysis indicated that individuals with potent viral inhibitory responses are rare, as was reported by others [24], and furthermore highlighted that responses to beneficial regions within the HIV proteome are both infrequent and subdominant. This is consistent with a previous study that showed infrequent targeting of epitopes in these regions in acute infection [32]. As spontaneous control of viraemia is itself a rare event, this provides further evidence that viral inhibitory activity in vitro accurately reflects immune control in vivo. It also raises questions as to whether long-term control or even clearance of infection can be achieved by vaccines that mimic priming by HIV. Responses elicited by the Ad5-HIV vaccine in HVTN 502 trial participants were shown previously to be limited in breadth, with a bias towards variable regions [2][7]. Our retrospective analysis of a subset of HVTN 502 vaccinees indicated preferential targeting of non-beneficial regions, which was concerning given that the Gag/Pol/Nef immunogen contained the majority of the previously described beneficial regions [14]. We observed a similar skewing of responses in HIV-positive subjects who received a therapeutic MVA vaccine encoding the immunogen, HIVA, which included 9 of the identified beneficial regions within Gag. Newer vaccine candidates such as Ad35-GRIN and Ad35-ENV, which comprise Gag, Reverse transcriptase, Integrase and Nef and Env sequences, induced responses to a median of one Gag epitope in HIV-uninfected healthy volunteers [40]. The common factor among these immunogens is the inclusion of full or near-full-length Gag sequences. A non-human primate study showed that full-length HIV immunogens induced responses to conserved regions that were of similar breadth to those elicited by non-native conserved region immunogens [52]; by contrast, Kulkarni et al. compared vaccination with p55 Gag and a conserved elements-only immunogen and showed better recognition of conserved elements epitopes with the latter approach [28,53]. Taken together, these observations highlight the need for vaccines to overcome natural immunodominance hierarchies in humans through the development of immunogens that focus responses on specific critical regions of the viral proteome. Additional refinements, such as inclusion of sequences that pre-empt predictable escape mutations, should also be considered [54]. Vaccine-mediated clearance of an AIDS virus infection in the non-human primate model was recently demonstrated for the first time with a persistent rhesus CMV SIV vaccine [55,56][57]. It is noteworthy that the responses elicited were unique in terms of their unprecedented breadth, absence of immunodominance and specificity for non-canonical viral epitopes, although the immunogen comprised entire proteins. While this may reflect unusual properties of the CMV vector and the specific mechanisms that contributed to virus eradication have yet to be resolved, such studies may provide vital lessons for human vaccine development.

In summary, these data provide several new insights that should inform HIV vaccine design. First, they suggest that induction of effective anti-HIV CD8+ T cell responses could be achieved with an immunogen comprising only a few selected regions of the viral proteome. In addition to the regions defined by Mothe et al., which were identified in chronically infected individuals, comprehensive analyses of responses that arise during acute / early HIV infection may yield viral targets that are critical to early and sustained control [32][58]. Secondly, we have identified a possible threshold for the magnitude of responses to these critical regions that should be attained in order to have a meaningful impact on viral replication. Our analysis of responses to vaccination with Ad5 Gag/Pol/Nef in a small subset of HVTN 502 subjects prior to HIV infection, together with other post-hoc studies, suggests that this is extremely unlikely to be achieved using immunogens that comprise full-length proteins. Exclusion of irrelevant decoy regions that when present, often induce immunodominant T cell responses, may be essential to prevent the development of such non-protective responses. Finally, our previous experience with potent heterologous viral vector combinations has shown that it is feasible to induce HIV-specific T cell responses in human subjects of the order of magnitude that we have proposed here [8]; rationally designed immunogens that exploit these vectors should be prioritised for clinical development.

## Methods

### Ethics statement

Approval was obtained from the Oxford Tropical Research Ethics Committee for analysis of anonymised PBMC samples that were made available to University of Oxford, UK by Fred Hutchinson Cancer Research Center via a Material Transfer Agreement (‘HVTN 502/Merck 023—HVTN 503 Ancillary Study’) following approval of the study by HVTN Protocol Committee. The PBMC samples were gathered and obtained from a collection held by HVTN. Viremic controllers (VC) were recruited at Duke University Medical Center with IRB approval and after obtaining written informed consent.

### Study participants

The HVTN 502 and 503 studies have been described previously [1][9]. PBMC sampled from 36 HIV-positive HVTN 502 and 503 participants who were still naïve to ART 12 months after HIV acquisition, with CD4+ cell counts >350 cells/μl, were provided through the HVTN 502 Oversight Committee. Plasma viral load data were provided by SCHARP and set-point was determined using the method described by Fellay et al. [59]. Participants’ characteristics are given in Table 1. Criteria for enrolment of VC were plasma viremia consistently <5000 copies/ml for at least one year and a CD4+ cell count >400 cells/μl in the absence of ART. However, one subject was included despite a CD4+ cell count <400 cells μl because of viral loads consistently <2280 copies/ml for 5 years prior to enrolment; this individual maintained viral loads <448 copies/ml during the study. Two subjects had transient viraemia >5000 copies/ml which was subsequently spontaneously controlled. Patients’ characteristics are given in Table 2. All VC had presumed clade B infection, due to the geographical location. Therapeutic vaccine trial participants were patients with chronic HIV infection, receiving effective ART for at least 12 months, with CD4+ cell counts >350 cells/μl, who received two intramuscular immunisations of MVA.HIVA 5x10^7^ pfu 4 weeks apart [34][60]. HLA typing was performed as described previously [6].

### Virus subtyping

Virus subtyping was performed by near full-length genome sequencing, as described previously [61] or by bulk sequencing of p17 Gag and analysis using REGA HIV-1 & 2 Automated Subtyping Tool (Version 2.0) [62][63].

### Virus isolates

HIV-1 isolates were obtained from the Programme EVA Centre for AIDS Reagents, National Institute for Biological Standards and Control (NIBSC), a centre of the Health Protection Agency, UK. The virus panel comprised two laboratory-adapted clade B isolates, BaL (CCR5-tropic) and IIIB (CXCR4-tropic) and three primary isolates, ES X-1936 (clade C, CCR5-tropic), 92UG029 (clade A, CCR5 / CXCR4 dual-tropic) and RW93024 (clade A, CXCR-tropic). All virus propagation was performed using primary CD4+ cells and 50% tissue culture infectious doses (TCID_50_) for each virus was calculated as described previously [64].

### Peptides

Clades B and C consensus peptides spanning the entire HIV proteome (15-mers overlapping by 11 amino acids) were obtained from the NIH Aids Reagent Programme. 10mg/ml stocks were stored at -80^°^C until required, then were diluted to generate working stocks. One or more 15-mer peptides that matched most closely the beneficial OLP described by Mothe et al. and the CE peptides described by Kulkarni et al. were selected for use in Elispot assays [14][28] (Tables 3–5).

### Viral inhibition assay

The viral inhibition assay has been described in detail elsewhere [16,65]. Briefly, CD8+ T cells were isolated from cryopreserved PBMC by magnetic bead selection (Miltenyi Biotec) and retained for use in IFN-γ Elispot assays. CD8-depleted cells (hereafter referred to as CD4+ T cells) were stimulated with PHA (5 μg/mL) in RPMI 1640 medium supplemented with 10% fetal calf serum (R10) for 3 days, washed, and infected with HIV-1 isolates at pre-determined optimal MOI (National Institute for Biological Standards and Control, United Kingdom). To assess viral inhibition, HIV-superinfected CD4+ T cells (5 × 10^4^) were cultured in triplicate in R10 with interleukin 2 (20 IU/mL) in 96-well round-bottomed plates, alone or together with unstimulated ex vivo CD8+ T cells, obtained by positive bead selection of PBMCs from a second freshly thawed vial on day 3. CD8+ T cells were confirmed as >98% pure by staining for CD3, CD8, and CD56. CD8+ and CD4+ T cells were co-cultured for 6 days for all virus isolates except clade A2, for which the peak of virus replication is attained after 3 days [65]. CD8+/CD4+ ratios of 2:1, 1:1 and 1:10 were tested, according to cell availability. On the day of harvest, cells were stained first with Aqua Live/Dead Fixable stain (Invitrogen), fixed with 1% paraformaldehyde/20 μg/mL lysolecithin at RT, permeabilized with cold 50% methanol followed by 0.1% Nonidet P-40, and finally stained with p24 antibody (KC-57-FITC; Beckman Coulter) and antibodies to CD3, CD4, and CD8 (conjugated to APC-Cy7, PerCP, and APC, respectively; BD Biosciences). Samples were acquired on a CyAn flow cytometer. Data were analyzed using FlowJo software. Antiviral suppressive activity was expressed as percentage inhibition and determined as follows: [(fraction of p24 + cells in CD4 + T cells cultured alone)–(fraction of p24 + in CD4+ T cells cultured with CD8+ cells)]/(fraction of p24 + cells in CD4 + T cells cultured alone) × 100.

### IFN-γ Elispot assay

Purified CD8+ T cells from the PBMC sample that was used to isolate CD4+ T cells for the viral inhibition assay were tested in IFN-γ Elispot assays with pools of beneficial or CE peptides (final concentration 2μg/ml) as described previously [16]. Mapping of responses to epitopes in the Gag component of the HIVA immunogen was performed using PBMC sampled pre- and 2 or 4 weeks post-vaccination, with overlapping 15-mer peptides (final concentration 4μg/ml) spanning the entire immunogen sequence, with confirmation using optimal 8–10-mer peptides where available [60]. Elispot assays with CD8-depleted PBMC were performed to confirm that these responses were CD8+ T cell-mediated. In selected assays, CD8+ T cells were recovered from the Elispot plate after overnight incubation with peptides, washed and cultured (2x10^6^/ml) in R10 medium (RPMI with 10% fetal calf serum) plus IL-7 (25ng/ml). Cultures were supplemented with IL-2 (1.8 x10^3^ units/ml) on day 3 and R10/IL-7/IL-2 medium was replaced on day 7. Cells were starved of IL-2 for 30 hours on day 10 and then used in cultured IFN-γ Elispot assays with individual peptides (2μg/ml).

### Intracellular cytokine assay

Intracellular cytokine staining was performed as described previously, typically at the second visit after HIV infection had been confirmed [66][2].

### Statistical analysis

Group comparisons were performed using the Mann Whitney test and correlations were investigated by determination of Spearman’s rank coefficient, using Graphpad Prism software, version 6. Models to explore predictors of inter-subject variation in viral inhibition by CD8+ T cells were tested using univariate and multivariable linear regression. Analyses were performed using SPSS version 22.

## Supporting Information

S1 Text Analysis of data from HVTN vaccinees only. (DOC)

S1 Fig CD8+ T cell antiviral inhibitory activity is inversely correlated with viral load set-point. Correlation between viral load set-point and CD8+ T cell-mediated inhibition of a clade-matched virus measured on day 6 of co-culture at a CD8+/CD4+ cell ratio of 2:1 in 28 HIV-positive HVTN 502 & 503 vaccinees (filled symbols) and placebos (open symbols) and 14 viraemic controllers (crosses) was assessed using Spearman rank test.
(TIF)

S1 Table Viral inhibition by CD8+ T cells. (DOCX)

S2 Table Consensus 15-mer peptides used to represent clade B beneficial regions. (DOCX)

S3 Table Consensus 15-mer peptides used to represent clade C beneficial regions. (DOCX)

S4 Table Consensus 15-mer peptides used to represent Gag p24 conserved elements. (DOCX)
