# Supplementary material for: Identification of Effective Subdominant Anti-HIV-1 CD8+ T Cells Within Entire Post-infection and Post-vaccination Immune Responses
Source: PLoS Pathog. 2015 Feb 27;11(2):e1004658. doi: 10.1371/journal.ppat.1004658 (PMC4344337; doi:10.1371/journal.ppat.1004658)
Supplement: S1 Text — (DOC) [file ppat.1004658.s001.doc]

**Supplementary Results – Analysis of data from HVTN vaccinees only**

**Figure 1**

B. Inhibition of clade-matched (B) virus at CD8/4 ratio of 2:1, excluding placebos from HVTN cohort

HVTN 502 vaccinees: median inhibition – 37%

HVTN 502 vaccinees vs. VCs, Mann-Whitney - p <0.0001

C. Inhibition of clade-matched (B) virus at CD8/4 ratio of 1:10, excluding placebos from HVTN cohort

HVTN 502 vaccinees: median inhibition – 0%

HVTN 502 vaccinees vs. VCs, Mann-Whitney - p <0.0001

D. Inhibition of clade-mismatched (C) virus – excluding placebos from HVTN cohort

HVTN 502 vaccinees: median inhibition at CD8/4 ratio of 2:1 – 10.5%

HVTN 502 vaccinees vs. VCs, Mann-Whitney – p = 0.005

**Figure 2**

C. Correlation between % inhibition (CD8/CD4 ratio of 2:1) and frequency of IFN-γ+ CD8+ T cells specific for beneficial regions within the HIV proteome in HVTN cohort, excluding placebos

Spearman r = 0.62, p = 0.007

D. Correlation between % inhibition (CD8/CD4 ratio of 2:1) and frequency of IFN-γ+ CD8+ T cells specific for beneficial regions within the HIV proteome in HVTN cohort (subjects without protective HLA class I alleles only), excluding placebos

Spearman r = 0.66, p = 0.02

F. Correlation between % inhibition (CD8/CD4 ratio 2:1) and response to conserved elements peptides in HVTN cohort, excluding placebos

i. All CE peptides: Spearman r = 0.49, p = 0.03

ii CE pool A peptides: Spearman r = 0.42, p = 0.07

iii. CE pool B peptides: Spearman r = 0.41, p = 0.08

**Figure 3**

B. Correlation between % inhibition and frequency of IFN-γ+ CD8+ T cells specific for the total HIV proteome, excluding placebos

Spearman r = -0.01, p = 0.98
