# Supplementary figures and images for: Identification of Effective Subdominant Anti-HIV-1 CD8+ T Cells Within Entire Post-infection and Post-vaccination Immune Responses
Source: PLoS Pathog. 2015 Feb 27;11(2):e1004658. doi: 10.1371/journal.ppat.1004658 (PMC4344337; doi:10.1371/journal.ppat.1004658)

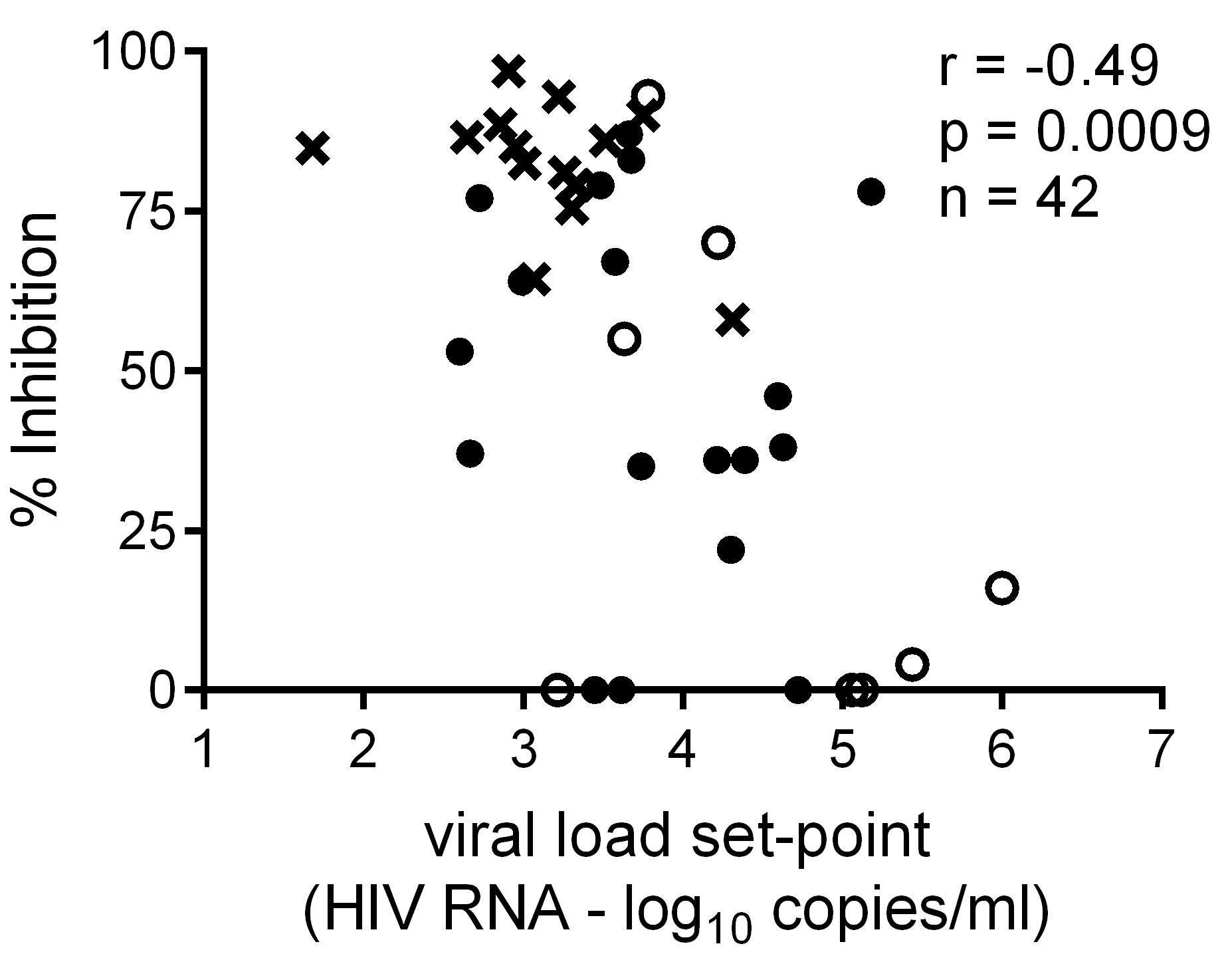

Supplement: S1 Fig — Correlation between viral load set-point and CD8+ T cell-mediated inhibition of a clade-matched virus measured on day 6 of co-culture at a CD8+/CD4+ cell ratio of 2:1 in 28 HIV-positive HVTN 502 & 503 vaccinees (filled symbols) and placebos (open symbols) and 14 viraemic controllers (crosses) was assessed using Spearman rank test. (TIF) [file ppat.1004658.s002.tif]
