# Supplementary material for: Identification of Effective Subdominant Anti-HIV-1 CD8+ T Cells Within Entire Post-infection and Post-vaccination Immune Responses
Source: PLoS Pathog. 2015 Feb 27;11(2):e1004658. doi: 10.1371/journal.ppat.1004658 (PMC4344337; doi:10.1371/journal.ppat.1004658)
Supplement: S1 Table — (DOCX) [file ppat.1004658.s003.docx]

| **Table S1 Viral inhibition by CD8+ T cells (%)** | | | | | | | | | | |
| --- | --- | --- | --- | --- | --- | --- | --- | --- | --- | --- |
|  | | | | | | | | | | |
| **Virus** | **BaL** | | **IIIB** | | **C** | | **A1** | | **A2** | |
| CD8/CD4 | 2:1 | 1:10 | 2:1 | 1:10 | 2:1 | 1:10 | 2:1 | 1:10 | 2:1 | 1:10 |
| **Subject ID** |  |  |  |  |  |  |  |  |  |  |
| VC3 | 84.8 | 60.1 | 79.7 | 31.7 | 24.8 | 4 | 30.3 | 14 | 41.7 | 2.6 |
| VC9 | 82.5 | 81.3 | 97.2 | 65.7 | 58.3 | 55.3 | 63.4 | 40.2 | 67.4 | 49.2 |
| VC10 | 79 | 68 | 94.4 | 69.3 | 87.5 | 64.9 | 76.3 | 33.7 | 78.6 | 37.2 |
| VC11 | 86 | 61 | 55 | 3.7 | 76 | 54 | 0 | 0 | 68.3 | 10.5 |
| VC13 | 81 | 47.8 | 73.6 | 49 | 30 | 10 | 41.3 | 27.8 | 83 | 51.7 |
| VC14 | 86.5 | 51.3 | 32 | 6.5 | 43.4 | 14.8 | 39.1 | 44.5 | 21.4 | 5.8 |
| VC16 | 92.9 | 73.2 | 96.1 | 58.9 | 67.4 | 52.2 | 56.4 | 45.5 | 42.5 | 30.2 |
| VC18 | 75.4 | 77.3 | 74 | 21.2 | 59.9 | 39.8 | 63.6 | 61.6 | 34 | 18 |
| VC20 | 96.9 | 88.3 | 98.9 | 81.2 | 96.8 | 61 | 94.2 | 66.5 | 90.2 | 20.1 |
| VC21 | 79 | 46 | 45 | 22 | 60.1 | 43.4 | 0 | 0 | 57.3 | 36.5 |
| VC22 | 88.5 | 59.3 | 2.6 | 0 | 15.6 | 9.9 | 46.7 | 31 | 81 | 38 |
| VC23 | 64.3 | 42.3 | 96.2 | 64.1 | 33.2 | 7.7 | 50 | 20 | 68.1 | 31.6 |
| VC24 | 85.1 | NA | 98.1 | 72.9 | 85.1 | 45.7 | 32.9 | 0 | NA | NA |
| VC25 | 90 | 62.2 | 64.8 | NA | 85.2 | 86.2 | 79.2 | 33.3 | 73.7 | 76.3 |
| 09896 | 0 | 0 | 37 | 13 | 0 | 0 | 2 | 0 | NA | NA |
| 10111 | 0 | 0 | 60 | 0 | 25 | 20 | 81 | 13 | NA | NA |
| 11613 | 55 | 37 | 51 | 7 | 33 | 0 | 24 | 6 | NA | NA |
| 09831 | 36 | 32 | NA | NA | NA | NA | NA | NA | NA | NA |
| 10276 | 0 | 0 | NA | NA | 11 | 20 | NA | NA | NA | NA |
| 13656 | 22 | 1 | NA | NA | NA | NA | NA | NA | NA | NA |
| 10338 | 35 | 0 | 0 | 7 | 0 | 0 | NA | NA | NA | NA |
| 13833 | 0 | 0 | 10 | 0 | 0 | 0 | NA | NA | NA | NA |
| 10110 | 79 | 55 | 95 | 20 | 10 | 0 | NA | NA | NA | NA |
| 11758 | 53* | NA | NA | NA | 0* | NA | NA | NA | NA | NA |
| 09754 | 67 | 0 | 64 | 30 | 43 | 27 | NA | NA | NA | NA |
| 13647 | 38 | 0 | NA | NA | 16 | 27 | NA | NA | NA | NA |
| 13937 | 53 | 0 | NA | NA | 6 | 32 | NA | NA | NA | NA |
| 11726 | 70 | 0 | 86 | NA | 57 | 55 | NA | NA | NA | NA |
| 13640 | 0 | NA | NA | NA | 0 | NA | NA | NA | NA | NA |
| 13728 | 77 | 0 | NA | NA | 83 | 44 | NA | NA | NA | NA |
| 11645 | 49* | 0 | 72* | 1 | 46* | 22 | NA | NA | NA | NA |
| 11860 | 46 | 19 | 45 | 42 | 65 | 9 | NA | NA | NA | NA |
| KC2010BQ | 27* | 0 | NA | NA | 51* | 0 | NA | NA | NA | NA |
| BC2024J4 | 33* | 0 | 0 | 0 | 32* | 33 | 38 | 33 | NA | NA |
| JC200YRJ | 41* | 0 | 48* | 5 | 63* | 2 | 2* | 0 | NA | NA |
| GA604NJH | 78 | 26 | 46* | 0 | 54* | 57 | 92 | 38 | NA | NA |
| D7Q03GZG | 47 | 44 | 3* | 6 | 36 | 20 | 3* | 19 | 13* | 30 |
| CAT02CMJ | 73 | NA | 43 | 34 | 87 | 26 | 67 | NA | NA | NA |
| FA603W25 | 40 | NA | 61 | 25 | 37 | 23 | 37 | 30 | 92 | 37 |
| JC203DLC | 19 | 13 | 30 | 0 | 78 | 36 | NA | NA | NA | NA |
| DC202KKR | 39 | 14 | 48* | NA | 93 | NA | NA | NA | NA | NA |
| F7Q04CBP | 29 | NA | NA | NA | 16 | NA | NA | NA | NA | NA |
| CC200ZOL | 6 | NA | NA | NA | 83 | NA | NA | NA | NA | NA |
| KA5060GL | 0 | 0 | NA | NA | 0 | 0 | 62 | 24 | 39 | 8 |
| K7Q04G4G | 0 | 0 | NA | NA | 4 | 28 | 7 | 19 | 74* | NA |
| JC2031JX | 46 | 14 | NA | NA | 58 | 15 | 71 | 44 | 81 | NA |
| DCG00GH2 | 0 | NA | NA | NA | 0 | 0 | NA | NA | NA | NA |
| JC203X51 | 42 | 50 | 47 | 0 | 64 | 40 | 32 | 0 | 50 | 38 |

Shading: blue – VC cohort; pink – HVTN 502; green – HVTN 503

* Tested at CD8+/CD4+ ratio of 1:1 if insufficient cells for 2:1 ratio
