# Supplementary material for: Identification of Effective Subdominant Anti-HIV-1 CD8+ T Cells Within Entire Post-infection and Post-vaccination Immune Responses
Source: PLoS Pathog. 2015 Feb 27;11(2):e1004658. doi: 10.1371/journal.ppat.1004658 (PMC4344337; doi:10.1371/journal.ppat.1004658)
Supplement: S2 Table — (DOCX) [file ppat.1004658.s004.docx]

| **Table S2** | | **Consensus 15-mer peptides used to represent clade B beneficial regions*** | | | | | | | | | | | | | | | | | | | | | | | | | | | | |  |
| --- | --- | --- | --- | --- | --- | --- | --- | --- | --- | --- | --- | --- | --- | --- | --- | --- | --- | --- | --- | --- | --- | --- | --- | --- | --- | --- | --- | --- | --- | --- | --- |
|  |  | |  |  |  | | | | | | | | | | | | | | | | | | | | | | | | | |  |
|  | **OLP #** | | **PR** | **Entropy** | **Sequence of beneficial region** | | | | | | | | | | | | | | | | | | | | | | | | | |  |
| **Gag**  **pool 1** | 31 | | 1.28 | 0.12 |  |  | I | A | P | G | Q | M | R | E | P | R | G | S | D | I | A |  |  |  |  |  |  |  |  |  |  |
|  |  | |  |  | G | P | - | - | - | - | - | - | - | - | - | - | - | - | - |  |  |  |  |  |  |  |  |  |  |  |  |
|  |  | |  |  |  |  |  |  | - | - | - | - | - | - | - | - | - | - | - | - | - | G | T |  |  |  |  |  |  |  |  |
|  | 34 | | 1.21 | 0.195 | S | T | L | Q | E | Q | I | G | W | M | T | N | N | P | P | I | P | V |  |  |  |  |  |  |  |  |  |
|  |  | |  |  | - | - | - | - | - | - | - | - | - | - | - | - | - | - | - |  |  |  |  |  |  |  |  |  |  |  |  |
|  |  | |  |  |  |  |  |  | - | - | - | - | - | - | - | - | - | - | - | - | - | - | G |  |  |  |  |  |  |  |  |
| **Gag**  **pool 2** | 6 | | 1.08 | 0.11 | A | S | R | E | L | E | R | F | A | V | N | P | G | L | L |  |  |  |  |  |  |  |  |  |  |  |  |
|  |  | |  |  | - | - | - | - | - | - | - | - | - | - | - | - | - | - | - |  |  |  |  |  |  |  |  |  |  |  |  |
|  | 48 | | 1.11 | 0.095 | A | C | Q | G | V | G | G | P | G | H | K | A | R | V | L | A | E | A |  |  |  |  |  |  |  |  |  |
|  |  | |  |  | - | - | - | - | - | - | - | - | - | - | - | - | - | - | - |  |  |  |  |  |  |  |  |  |  |  |  |
|  |  | |  |  |  |  |  |  | - | - | - | - | - | - | - | - | - | - | - | - | - | - | M |  |  |  |  |  |  |  |  |
| **Gag**  **pool 3** | 3 | | 1.05 | 0.24 | E | K | I | R | L | R | P | G | G | K | K | K | Y | K | L | K | H | I |  |  |  |  |  |  |  |  |  |
|  |  | |  |  | - | - | - | - | - | - | - | - | - | - | - | - | - | - | - |  |  |  |  |  |  |  |  |  |  |  |  |
|  |  | |  |  |  |  |  |  | - | - | - | - | - | - | - | - | - | - | - | - | - | - | V |  |  |  |  |  |  |  |  |
|  | 7 | | 1.04 | 0.23 |  | E | R | F | A | V | N | P | G | L | L | E | T | S | E | G | C | R |  |  |  |  |  |  |  |  |  |
|  |  | |  |  | - | - | - | - | - | - | - | - | - | - | - | - | - | - | - |  |  |  |  |  |  |  |  |  |  |  |  |
|  |  | |  |  |  |  |  |  | - | - | - | - | - | - | - | - | - | - | - | - | - | - | Q |  |  |  |  |  |  |  |  |
|  | 10 | | 1.09 | 0.285 |  |  | Q | L | Q | P | S | L | Q | T | G | S | E | E | L | R | S | L | Y |  |  |  |  |  |  |  |  |
|  |  | |  |  | L | G | - | - | - | - | - | - | - | - | - | - | - | - | - |  |  |  |  |  |  |  |  |  |  |  |  |
|  |  | |  |  |  |  |  |  | - | - | - | - | - | - | - | - | - | - | - | - | - | - | - |  |  |  |  |  |  |  |  |
|  | 12 | | 1.04 | 0.365 | S | L | Y | N | T | V | A | T | L | Y | C | V | H | Q | R | I | E | V |  |  |  |  |  |  |  |  |  |
|  |  | |  |  | - | - | - | - | - | - | - | - | - | - | - | - | - | - | - |  |  |  |  |  |  |  |  |  |  |  |  |
|  |  | |  |  |  |  |  |  | - | - | - | - | - | - | - | - | - | - | - | - | - | - | K |  |  |  |  |  |  |  |  |
|  | 23 | | 1.05 | 0.1 |  |  | A | F | S | P | E | V | I | P | M | F | S | A | L | S | E | G | A |  |  |  |  |  |  |  |  |
|  |  | |  |  | E | K | - | - | - | - | - | - | - | - | - | - | - | - | - |  |  |  |  |  |  |  |  |  |  |  |  |
|  |  | |  |  |  |  |  |  | - | - | - | - | - | - | - | - | - | - | - | - | - | - | - |  |  |  |  |  |  |  |  |
|  | 60 | | 1.08 | 0.36 |  |  | G | K | I | W | P | S | H | K | G | R | P | G | N | F | L | Q | S | R |  |  |  |  |  |  |  |
|  |  | |  |  | F | L | - | - | - | - | - | - | - | - | - | - | - | - | - |  |  |  |  |  |  |  |  |  |  |  |  |
|  |  | |  |  |  |  |  |  | - | - | - | - | - | - | - | - | - | - | - | - | - | - | - |  |  |  |  |  |  |  |  |
|  |  | |  |  |  |  |  |  |  |  |  | - | - | - | - | - | - | - | - | - | - | - | - | - | P | E | P |  |  |  |  |
| **Pol**  **pool 1** | 160 | | 1.28 | 0.335 | F | I | K | V | R | Q | Y | D | Q | I | L | I | E | I | C | G | H | K |  |  |  |  |  |  |  |  |  |
|  |  | |  |  | - | - | - | - | - | - | - | - | - | - | - | - | - | - | - |  |  |  |  |  |  |  |  |  |  |  |  |
|  |  | |  |  |  |  |  |  | - | - | - | - | - | - | - | - | - | - | - | - | - | - | A |  |  |  |  |  |  |  |  |
|  | 171 | | 1.39 | 0.16 | L | V | E | I | C | T | E | M | E | K | E | G | K | I | S | K | I |  |  |  |  |  |  |  |  |  |  |
|  |  | |  |  | - | - | - | - | - | - | - | - | - | - | - | - | - | - | - |  |  |  |  |  |  |  |  |  |  |  |  |
|  |  | |  |  |  |  |  |  | - | - | - | - | - | - | - | - | - | - | - | - | - | G | P |  |  |  |  |  |  |  |  |
|  | 195 | | 1.22 | 0.3 | L | R | W | G | F | T | T | P | D | K | K | H | Q | K | E | P | P | F |  |  |  |  |  |  |  |  |  |
|  |  | |  |  | - | - | - | - | - | - | - | - | - | - | - | - | - | - | - |  |  |  |  |  |  |  |  |  |  |  |  |
|  |  | |  |  |  |  |  |  | - | - | - | - | - | - | - | - | - | - | - | - | - | - | L |  |  |  |  |  |  |  |  |
| **Pol**  **pool 2** | 161 | | 1.15 | 0.345 | Q | I | L | I | E | I | C | G | H | K | A | I | G | T | V | L | V |  |  |  |  |  |  |  |  |  |  |
|  |  | |  |  | - | - | - | - | - | - | - | - | - | - | - | - | - | - | - |  |  |  |  |  |  |  |  |  |  |  |  |
|  |  | |  |  |  |  |  |  | - | - | - | - | - | - | - | - | - | - | - | - | - | G | P |  |  |  |  |  |  |  |  |
|  | 196 | | 1.14 | 0.085 | D | K | K | H | Q | K | E | P | P | F | L | W | M | G | Y | E | L | H |  |  |  |  |  |  |  |  |  |
|  |  | |  |  | - | - | - | - | - | - | - | - | - | - | - | - | - | - | - |  |  |  |  |  |  |  |  |  |  |  |  |
|  |  | |  |  |  |  |  |  | - | - | - | - | - | - | - | - | - | - | - | - | - | - | P |  |  |  |  |  |  |  |  |
|  | 269 | | 1.19 | 0.135 | T | K | E | L | Q | K | Q | I | T | K | I | Q | N | F | R | V | Y | Y |  |  |  |  |  |  |  |  |  |
|  |  | |  |  | - | - | - | - | - | - | - | - | - | - | - | - | - | - | - |  |  |  |  |  |  |  |  |  |  |  |  |
|  |  | |  |  |  |  |  |  | - | - | - | - | - | - | - | - | - | - | - | - | - | - | R |  |  |  |  |  |  |  |  |
|  | 276 | | 1.19 | 0.055 | K | I | I | R | D | Y | G | K | Q | M | A | G | D | D | C | V | A |  |  |  |  |  |  |  |  |  |  |
|  |  | |  |  | - | - | - | - | - | - | - | - | - | - | - | - | - | - | - |  |  |  |  |  |  |  |  |  |  |  |  |
|  |  | |  |  |  |  |  |  | - | - | - | - | - | - | - | - | - | - | - | - | - | S | R |  |  |  |  |  |  |  |  |
| **Pol**  **pool 3** | 159 | | 1.09 | 0.19 | K | M | I | G | G | I | G | G | F | I | K | V | R | Q | Y | D | Q | I |  |  |  |  |  |  |  |  |  |
|  |  | |  |  | - | - | - | - | - | - | - | - | - | - | - | - | - | - | - |  |  |  |  |  |  |  |  |  |  |  |  |
|  |  | |  |  |  |  |  |  | - | - | - | - | - | - | - | - | - | - | - | - | - | - | L |  |  |  |  |  |  |  |  |
|  | 163 | | 1.06 | 0.16 |  |  |  | L | V | G | P | T | P | V | N | I | I | G | R | N | L | L | T | Q | I |  |  |  |  |  |  |
|  |  | |  |  | G | T | V | - | - | - | - | - | - | - | - | - | - | - | - |  |  |  |  |  |  |  |  |  |  |  |  |
|  |  | |  |  |  |  |  |  | - | - | - | - | - | - | - | - | - | - | - | - | - | - | - |  |  |  |  |  |  |  |  |
|  |  | |  |  |  |  |  |  |  |  |  |  | - | - | - | - | - | - | - | - | - | - | - | - | - | G | C |  |  |  |  |
|  | 210 | | 1.07 | 0.24 |  |  |  |  |  |  | E | I | Q | K | Q | G | Q | G | Q | W | T | Y | Q | I | Y |  |  |  |  |  |  |
|  |  | |  |  | S | K | D | L | I | A | - | - | - | - | - | - | - | - | - |  |  |  |  |  |  |  |  |  |  |  |  |
|  |  | |  |  |  |  |  |  |  |  | - | - | - | - | - | - | - | - | - | - | - | - | - | - | - |  |  |  |  |  |  |
|  | 270 | | 1.07 | 0.125 | T | K | I | Q | N | F | R | V | Y | Y | R | D | S | R | D | P | L | W |  |  |  |  |  |  |  |  |  |
|  |  | |  |  | - | - | - | - | - | - | - | - | - | - | - | - | - | - | - |  |  |  |  |  |  |  |  |  |  |  |  |
|  |  | |  |  |  |  |  |  | - | - | - | - | - | - | - | - | - | - | - | - | - | - | K |  |  |  |  |  |  |  |  |
|  | 271 | | 1.03 | 0.09 | Y | Y | R | D | S | R | D | P | L | W | K | G | P | A | K | L | L | W |  |  |  |  |  |  |  |  |  |
|  |  | |  |  | - | - | - | - | - | - | - | - | - | - | - | - | - | - | - |  |  |  |  |  |  |  |  |  |  |  |  |
|  |  | |  |  |  |  |  |  | - | - | - | - | - | - | - | - | - | - | - | - | - | - | K |  |  |  |  |  |  |  |  |
| **Vif**  **pool 1** | 405 | | 1.08 | 0.53 | V | K | H | H | M | Y | I | S | G | K | A | K | G | W | F | Y | R | H |  |  |  |  |  |  |  |  |  |
|  |  | |  |  | - | - | - | - | - | - | - | - | - | - | - | - | - | - | - | - | - | - |  |  |  |  |  |  |  |  |  |
|  | 406 | | 1.08 | 0.54 |  |  |  |  | G | K | A | K | G | W | F | Y | R | H | H | Y | E | S | T | H | P | R |  |  |  |  |  |
|  |  | |  |  | M | Y | I | S | - | - | - | - | - | - | - | - | - | - | - |  |  |  |  |  |  |  |  |  |  |  |  |
|  |  | |  |  |  |  |  |  | - | - | - | - | - | - | - | - | - | - | - | - | - | - | - |  |  |  |  |  |  |  |  |
|  |  | |  |  |  |  |  |  |  |  |  |  | - | - | - | - | - | - | - | - | - | - | - | - | - | - | I |  |  |  |  |
|  | 424 | | 1.14 | 0.25 |  |  | T | K | L | T | E | D | R | W | N | K | P | Q | K | T | K | G | H | R |  |  |  |  |  |  |  |
|  |  | |  |  | S | V | - | - | - | - | - | - | - | - | - | - | - | - | - |  |  |  |  |  |  |  |  |  |  |  |  |
|  |  | |  |  |  |  |  |  | - | - | - | - | - | - | - | - | - | - | - | - | - | - | - |  |  |  |  |  |  |  |  |
|  |  | |  |  |  |  |  |  |  |  |  |  | - | - | - | - | - | - | - | - | - | - | - | - | G | S | H |  |  |  |  |
| **Nef** | 75 | | 1.11 | 0.395 | W | L | E | A | Q | E | E | E | E | V | G | F | P | V | R | P | Q | V |  |  |  |  |  |  |  |  |  |
|  |  | |  |  | - | - | - | - | - | - | - | - | - | - | - | - | - | - | - |  |  |  |  |  |  |  |  |  |  |  |  |
|  |  | |  |  |  |  |  |  | - | - | - | - | - | - | - | - | - | - | - | - | - | - | P |  |  |  |  |  |  |  |  |

* The 18-mer OLP sequence and its protective ratio (PR) defined by Mothe et al. is shown, with corresponding overlapping clade B 15-mer peptide(s) used for HVTN 502 subjects indicated by dashed lines.
