# Supplementary material for: Identification of Effective Subdominant Anti-HIV-1 CD8+ T Cells Within Entire Post-infection and Post-vaccination Immune Responses
Source: PLoS Pathog. 2015 Feb 27;11(2):e1004658. doi: 10.1371/journal.ppat.1004658 (PMC4344337; doi:10.1371/journal.ppat.1004658)
Supplement: S3 Table — (DOCX) [file ppat.1004658.s005.docx]

| **Table S3** | | **Consensus 15-mer peptides used to represent clade C beneficial regions*** | | | | | | | | | | | | | | | | | | | | | | | | | | | | |
| --- | --- | --- | --- | --- | --- | --- | --- | --- | --- | --- | --- | --- | --- | --- | --- | --- | --- | --- | --- | --- | --- | --- | --- | --- | --- | --- | --- | --- | --- | --- |
|  |  | |  |  |  | | | | | | | | | | | | | | | | | | | | | | | | | |
|  | **OLP #** | | **PR** | **Entropy** | **Sequence of beneficial region** | | | | | | | | | | | | | | | | | | | | | | | | | |
| **Gag pool 1** | 7 | | 1.25 | 0.23 |  | E | R | F | A | L | N | P | G | L | L | E | T | S | E | G | C | K |  |  |  |  |  |  |  |  |
|  |  | |  |  | L | - | - | - | - | - | - | - | - | - | - | - | - | - | - | - | - | - |  |  |  |  |  |  |  |  |
|  | 26 | | 1.23 | 0.08 | N | T | M | L | N | T | V | G | G | H | Q | A | A | M | Q | M | L | K |  |  |  |  |  |  |  |  |
|  |  | |  |  | - | - | - | - | - | - | - | - | - | - | - | - | - | - | - |  |  |  |  |  |  |  |  |  |  |  |
|  |  | |  |  |  |  |  |  | - | - | - | - | - | - | - | - | - | - | - | - | - | - | E |  |  |  |  |  |  |  |
|  | 39 | | 1.34 | 0.15 |  |  |  |  |  | S | I | L | D | I | K | Q | G | P | K | E | P | F | R | D | Y | V |  |  |  |  |
|  |  | |  |  | M | Y | S | P | V | - | - | - | - | - | - | - | - | - | - | - | - | - |  |  |  |  |  |  |  |  |
|  |  | |  |  |  |  |  |  |  |  |  |  | - | - | - | - | - | - | - | - | - | - | - | - | - | - | D | R | F | F |
|  |  | |  |  |  |  |  |  |  |  |  |  |  |  |  |  |  |  |  |  |  |  |  |  |  |  |  |  |  |  |
| **Gag pool 2** | 6 | | 1.22 | 0.24 |  |  |  |  | A | S | R | E | L | E | R | F | A | L | N | P | G | L | L |  |  |  |  |  |  |  |
|  |  | |  |  | H | L | V | W | - | - | - | - | - | - | - | - | - | - | - | - | - | - |  |  |  |  |  |  |  |  |
|  |  | |  |  |  |  |  |  |  |  |  |  | - | - | - | - | - | - | - | - | - | - | - | E | T | S | E | G | C | K |
|  | 60 | | 1.21 | 0.36 |  |  | G | K | I | W | P | S | H | K | G | R | P | G | N | F | L | Q | S | R |  |  |  |  |  |  |
|  |  | |  |  | F | L | - | - | - | - | - | - | - | - | - | - | - | - | - |  |  |  |  |  |  |  |  |  |  |  |
|  |  | |  |  |  |  |  |  | - | - | - | - | - | - | - | - | - | - | - | - | - | - | - |  |  |  |  |  |  |  |
|  |  | |  |  |  |  |  |  |  |  |  |  | - | - | - | - | - | - | - | - | - | - | - | - | P | E | P |  |  |  |
|  | 63 | | 1.21 | 0.84 |  |  |  | T | A | P | P | A | E | S | F | R | F | E | E | T | T | P | A | P | K |  |  |  |  |  |
|  |  | |  |  | P | E | P | - | - | - | - | - | - | - | - | - | - | - | - | - | - | - |  |  |  |  |  |  |  |  |
|  |  | |  |  |  |  |  |  |  |  |  |  | - | - | - | - | - | - | - | - | - | - | - | - | - | Q | E | P | K | D |
|  |  | |  |  |  |  |  |  |  |  |  |  |  |  |  |  |  |  |  |  |  |  |  |  |  |  |  |  |  |  |
| **Gag pool 3** | 22 | | 1.18 | 0.14 |  |  |  |  |  |  | W | V | K | V | I | E | E | K | A | F | S | P | E | V | I | P | M | F |  |  |
|  |  | |  |  | P | R | T | L | N | A | - | - | - | - | - | - | - | - | - | - | - |  |  |  |  |  |  |  |  |  |
|  |  | |  |  |  |  |  |  |  |  |  | - | - | - | - | - | - | - | - | - | - | - | - | - | - | - | - | - | T |  |
|  | 31 | | 1.2 | 0.15 |  |  | I | A | P | G | Q | M | R | E | P | R | G | S | D | I | A |  |  |  |  |  |  |  |  |  |
|  |  | |  |  | G | P | - | - | - | - | - | - | - | - | - | - | - | - | - |  |  |  |  |  |  |  |  |  |  |  |
|  |  | |  |  |  |  |  |  | - | - | - | - | - | - | - | - | - | - | - | - | - | G | T |  |  |  |  |  |  |  |
|  | 55 | | 1.19 | 0.14 |  |  |  | H | I | A | R | N | C | R | A | P | R | K | K | G | C | W | K |  |  |  |  |  |  |  |
|  |  | |  |  | K | E | G | - | - | - | - | - | - | - | - | - | - | - | - | - | - | - |  |  |  |  |  |  |  |  |
|  |  | |  |  |  |  |  |  |  |  |  |  | - | - | - | - | - | - | - | - | - | - | - | C | G | K | E | G | H | Q |
|  |  | |  |  |  |  |  |  |  |  |  |  |  |  |  |  |  |  |  |  |  |  |  |  |  |  |  |  |  |  |
| **Gag pool 4** | 27 | | 1.16 | 0.08 |  |  |  |  | G | G | H | Q | A | A | M | Q | M | L | K | D | T | I | N | E | E | A |  |  |  |  |
|  |  | |  |  | L | N | T | V | - | - | - | - | - | - | - | - | - | - | - | - | - | - |  |  |  |  |  |  |  |  |
|  |  | |  |  |  |  |  |  |  |  |  | - | - | - | - | - | - | - | - | - | - | - | - | - | - | - | A | E | W | D |
|  | 37 | | 1.17 | 0.08 |  |  |  |  |  | W | I | I | L | G | L | N | K | I | V | R | M | Y | S | P | V | S | I |  |  |  |
|  |  | |  |  | D | I | Y | K | R | - | - | - | - | - | - | - | - | - | - | - | - | - |  |  |  |  |  |  |  |  |
|  |  | |  |  |  |  |  |  |  |  |  |  | - | - | - | - | - | - | - | - | - | - | - | - | - | - | - | L | D | I |
|  | 59 | | 1.16 | 0.18 | R | Q | A | N | F | L | G | K | I | W | P | S | H | K | G | R |  |  |  |  |  |  |  |  |  |  |
|  |  | |  |  | - | - | - | - | - | - | - | - | - | - | - | - | - | - | - | - |  |  |  |  |  |  |  |  |  |  |
|  |  | |  |  |  |  |  |  |  |  |  |  |  |  |  |  |  |  |  |  |  |  |  |  |  |  |  |  |  |  |
| **Gag pool 5** | 3 | | 1.09 | 0.31 | E | K | I | R | L | R | P | G | G | K | K | H | Y | M | L | K | H | L |  |  |  |  |  |  |  |  |
|  |  | |  |  | - | - | - | - | - | - | - | - | - | - | - | - | - | - | - | - | - | - |  |  |  |  |  |  |  |  |
|  | 42 | | 1.09 | 0.19 |  |  |  |  | L | R | A | E | Q | A | T | Q | D | V | K | N | W | M | T | D | T | L |  |  |  |  |
|  |  | |  |  | F | F | K | T | - | - | - | - | - | - | - | - | - | - | - | - | - | - |  |  |  |  |  |  |  |  |
|  |  | |  |  |  |  |  |  |  |  |  | - | - | - | - | - | - | - | - | - | - | - | - | - | - | - | L | V | Q | N |
|  | 33 | | 1.13 | 0.17 |  |  |  |  |  |  | S | D | I | A | G | T | T | S | T | L | Q | E | Q | I | A | W | M |  |  |  |
|  |  | |  |  | M | R | E | P | R | G | - | - | - | - | - | - | - | - | - | - | - | - |  |  |  |  |  |  |  |  |
|  |  | |  |  |  |  |  |  |  |  |  | - | - | - | - | - | - | - | - | - | - | - | - | - | - | - | - | T | S | N |
|  |  | |  |  |  |  |  |  |  |  |  |  |  |  |  |  |  |  |  |  |  |  |  |  |  |  |  |  |  |  |
| **Gag pool 6** | 29 | | 1.07 | 0.23 | A | A | E | W | D | R | L | H | P | V | H | A | G | P | I | A |  |  |  |  |  |  |  |  |  |  |
|  |  | |  |  | - | - | - | - | - | - | - | - | - | - | - | - | - | - | - |  |  |  |  |  |  |  |  |  |  |  |
|  |  | |  |  |  |  |  | - | - | - | - | - | - | - | - | - | - | - | - | - | P | G | Q |  |  |  |  |  |  |  |
|  | 41 | | 1.07 | 0.17 |  |  |  |  | Y | V | D | R | F | F | K | T | L | R | A | E | Q | A | T | Q | D | V |  |  |  |  |
|  |  | |  |  | P | F | R | D | - | - | - | - | - | - | - | - | - | - | - | - | - | - |  |  |  |  |  |  |  |  |
|  |  | |  |  |  |  |  |  |  |  |  |  | - | - | - | - | - | - | - | - | - | - | - | - | - | - | K | N | W | M |
|  | 25 | | 1.06 | 0.12 |  | G | A | T | P | Q | D | L | N | T | M | L | N | T | V | G | G | H |  |  |  |  |  |  |  |  |
|  |  | |  |  | E | - | - | - | - | - | - | - | - | - | - | - | - | - | - |  |  |  |  |  |  |  |  |  |  |  |
|  |  | |  |  |  |  |  |  | - | - | - | - | - | - | - | - | - | - | - | - | - | - | Q |  |  |  |  |  |  |  |
|  |  | |  |  |  |  |  |  |  |  |  |  |  |  |  |  |  |  |  |  |  |  |  |  |  |  |  |  |  |  |
| **Pol pool 1** | 181 | | 1.19 | 0.14 |  |  |  |  |  |  |  | L | D | V | G | D | A | Y | F | S | V | P | L | D | E | D | F | R | K |  |
|  |  | |  |  | K | K | K | S | V | T | V | - | - | - | - | - | - | - | - | - | - | - |  |  |  |  |  |  |  |  |
|  |  | |  |  |  |  |  |  |  |  |  | - | - | - | - | - | - | - | - | - | - | - | - | - | - | - | - | - | - | Y |
|  | 199 | | 1.2 | 0.16 |  |  | T | V | Q | P | I | Q | L | P | E | K | D | S | W | T | V | N | D | I |  |  |  |  |  |  |
|  |  | |  |  |  |  | - | - | - | - | - | - | - | - | - | - | - | - | - | - | - | - | - | - |  |  |  |  |  |  |
|  | 216 | | 1.09 | 0.24 |  |  |  |  |  |  |  | Q | K | I | A | M | E | S | I | V | I | W | G | K | T | P | K | F | R |  |
|  |  | |  |  | K | Q | L | T | E | A | V | - | - | - | - | - | - | - | - | - | - | - |  |  |  |  |  |  |  |  |
|  |  | |  |  |  |  |  |  |  |  |  |  | - | - | - | - | - | - | - | - | - | - | - | - | - | - | - | - | - | L |
|  |  | |  |  |  |  |  |  |  |  |  |  |  |  |  |  |  |  |  |  |  |  |  |  |  |  |  |  |  |  |
| **Vif** | 417 | | 1.5 | 0.26 |  | C | F | A | D | S | A | I | R | K | A | I | L | G | H | I | V |  |  |  |  |  |  |  |  |  |
|  |  | |  |  | D | - | - | - | - | - | - | - | - | - | - | - | - | - | - | - | - | I |  |  |  |  |  |  |  |  |

* The 18-mer OLP sequence and its protective ratio (PR) defined by Mothe et al. [14] is shown, with corresponding overlapping clade C 15-mer peptide(s) used for HVTN503 subjects indicated by dashed lines.
