# Supplementary material for: Identification of Effective Subdominant Anti-HIV-1 CD8+ T Cells Within Entire Post-infection and Post-vaccination Immune Responses
Source: PLoS Pathog. 2015 Feb 27;11(2):e1004658. doi: 10.1371/journal.ppat.1004658 (PMC4344337; doi:10.1371/journal.ppat.1004658)
Supplement: S4 Table — (DOCX) [file ppat.1004658.s006.docx]

| **Table S4** | | **Consensus 15-mer peptides used to represent Gag p24 conserved elements*** | | | | | | | | | | | | | | | | | | | | | | | | | | | | | | | | | |
| --- | --- | --- | --- | --- | --- | --- | --- | --- | --- | --- | --- | --- | --- | --- | --- | --- | --- | --- | --- | --- | --- | --- | --- | --- | --- | --- | --- | --- | --- | --- | --- | --- | --- | --- | --- |
|  |  | |  |  | | | | | | | | | | | | | | | | | | | | | | | | | | | | | | |  |
|  | **OLP #** | | **Entropy** | **Sequence of Gag p24 conserved elements** | | | | | | | | | | | | | | | | | | | | | | | | | | | | | | |  |
| **Pool A** | CE1 | | 0.21 |  |  | I | S | P | R | T | L | N | A | W | V | K | V |  |  |  |  |  |  |  |  |  |  |  |  |  |  |  |  |  |  |
|  |  | |  | Q | A | - | - | - | - | - | - | - | - | - | - | - | - | V |  |  |  |  |  |  |  |  |  |  |  |  |  |  |  |  |  |
|  | CE2 | | 0.13 |  |  |  | V | I | P | M | F | S | A | L | S | E | G | A | T | P | Q | D | L | N |  |  |  |  |  |  |  |  |  |  |  |
|  |  | |  | S | P | E | - | - | - | - | - | - | - | - | - | - | - | - |  |  |  |  |  |  |  |  |  |  |  |  |  |  |  |  |  |
|  |  | |  |  |  |  | - | - | - | - | - | - | - | - | - | - | - | - | - | - | - | - |  |  |  |  |  |  |  |  |  |  |  |  |  |
|  |  | |  |  |  |  |  |  |  |  |  | - | - | - | - | - | - | - | - | - | - | - | - | - | T | M |  |  |  |  |  |  |  |  |  |
|  | CE3 | | 0.11 |  |  | V | G | G | H | Q | A | A | M | Q | M | L | K | D | T | I | N | E | E | A | A | E | W | D | R |  |  |  |  |  |  |
|  |  | |  | N | T | - | - | - | - | - | - | - | - | - | - | - | - | - |  |  |  |  |  |  |  |  |  |  |  |  |  |  |  |  |  |
|  |  | |  |  |  |  |  | - | - | - | - | - | - | - | - | - | - | - | - | - | - | - |  |  |  |  |  |  |  |  |  |  |  |  |  |
|  |  | |  |  |  |  |  |  |  |  | - | - | - | - | - | - | - | - | - | - | - | - | - | - | - | - |  |  |  |  |  |  |  |  |  |
|  |  | |  |  |  |  |  |  |  |  |  |  |  |  |  | - | - | - | - | - | - | - | - | - | - | - | - | - | - | L |  |  |  |  |  |
|  | CE7 | | 0.09 |  |  | L | E | E | M | M | T | A | C | Q | G | V | G | G | P | S | H | K |  |  |  |  |  |  |  |  |  |  |  |  |  |
|  |  | |  | A | T | - | - | - | - | - | - | - | - | - | - | - | - | - |  |  |  |  |  |  |  |  |  |  |  |  |  |  |  |  |  |
|  |  | |  |  |  |  | - | - | - | - | - | - | - | - | - | - | - | - | - | G | - | - |  |  |  |  |  |  |  |  |  |  |  |  |  |
| **Pool B** | CE4 | | 0.11 |  |  |  |  |  |  |  |  |  |  | P | R | G | S | D | I | A | G | T | T | S | T | L | Q | E | Q | I | G | W |  |  |  |
|  |  | |  | G | P | I | A | P | G | Q | M | R | E | - | - | - | - | - |  |  |  |  |  |  |  |  |  |  |  |  |  |  |  |  |  |
|  |  | |  |  |  |  |  | P | G | Q | M | R | E | - | - | - | - | - | - | - | - | - |  |  |  |  |  |  |  |  |  |  |  |  |  |
|  |  | |  |  |  |  |  |  |  |  |  | R | E | - | - | - | - | - | - | - | - | - | - | - | - | - |  |  |  |  |  |  |  |  |  |
|  |  | |  |  |  |  |  |  |  |  |  |  |  |  |  | - | - | - | - | - | - | - | - | - | - | - | - | - | - | - |  |  |  |  |  |
|  |  | |  |  |  |  |  |  |  |  |  |  |  |  |  |  |  |  |  | - | - | - | - | - | - | - | - | - | - | - | - | - | M | T |  |
|  | CE5 | | 0.09 |  |  | K | R | W | I | I | L | G | L | N | K | I | V | R | M | Y | S | P | V | S | I |  |  |  |  |  |  |  |  |  |  |
|  |  | |  | I | Y | - | - | - | - | - | - | - | - | - | - | - | - | - |  |  |  |  |  |  |  |  |  |  |  |  |  |  |  |  |  |
|  |  | |  |  |  |  |  | - | - | - | - | - | - | - | - | - | - | - | - | - | - | - |  |  |  |  |  |  |  |  |  |  |  |  |  |
|  |  | |  |  |  |  |  |  |  |  |  | - | - | - | - | - | - | - | - | - | - | - | T | - | - | L |  |  |  |  |  |  |  |  |  |
|  | CE6 | | 0.15 |  |  |  | Y | V | D | R | F | F | K | T | L | R | A | E | Q | A |  |  |  |  |  |  |  |  |  |  |  |  |  |  |  |
|  |  | |  | F | R | D | - | - | - | - | - | Y | - | - | - | - | - | - |  |  |  |  |  |  |  |  |  |  |  |  |  |  |  |  |  |
|  |  | |  |  |  |  |  | - | - | - | - | Y | - | - | - | - | - | - | - | - | S | Q |  |  |  |  |  |  |  |  |  |  |  |  |  |

* The conserved element sequence in Gag p24 defined by Rolland et al. is shown, with corresponding overlapping clade B 15-mer peptides used indicated by dashed lines.
